# Proton Quantum Tunneling: Influence and Relevance to Acidosis-Induced Cardiac Arrhythmias/Cardiac Arrest

**DOI:** 10.3390/pathophysiology28030027

**Published:** 2021-09-03

**Authors:** Omar Ababneh, Abdallah Barjas Qaswal, Ahmad Alelaumi, Lubna Khreesha, Mujahed Almomani, Majdi Khrais, Oweiss Khrais, Ahmad Suleihat, Shahed Mutleq, Yazan Al-olaimat, Sager Nawafleh

**Affiliations:** 1 Department of Anesthesia and Intensive Care, School of Medicine, The University of Jordan, Amman 11942, Jordan; omar.ababneh@ju.edu.jo; 2 Department of Internal Medicine, School of Medicine, The University of Jordan, Amman 11942, Jordan; 3 Department of Orthopedic Surgery, School of Medicine, The University of Jordan, Amman 11942, Jordan; ahmadfouad999@gmail.com; 4 Department of Special Surgery, School of Medicine, The University of Jordan, Amman 11942, Jordan; l.khreesha@ju.edu.jo; 5 Department of General Surgery, School of Medicine, The University of Jordan, Amman 11942, Jordan; mujahedalmomany@gmail.com (M.A.); majdikhrais@gmail.com (M.K.); Oweis.khrais@yahoo.com (O.K.); Ahmad_slaihat@yahoo.com (A.S.); 6 Department of Family Medicine, School of Medicine, The University of Jordan, Amman 11942, Jordan; shahdhasan1@gmail.com; 7 Department of Neurosurgery, School of Medicine, The University of Jordan, Amman 11942, Jordan; olimat_1989@yahoo.com; 8 Department of Anesthesia and Intensive Care Unit, The Hashemite University, Zarqa 13115, Jordan; Sager@hu.edu.jo

**Keywords:** quantum tunneling, proton, acidosis, quantum biology, quantum conductance, voltage-gated channels, arrhythmias

## Abstract

Acidosis and its associated pathologies predispose patients to develop cardiac arrhythmias and even cardiac arrest. These arrhythmias are assumed to be the result of membrane depolarization, however, the exact mechanism of depolarization during acidosis is not well defined. In our study, the model of quantum tunneling of protons is used to explain the membrane depolarization that occurs during acidosis. It is found that protons can tunnel through closed activation and inactivation gates of voltage-gated sodium channels Nav1.5 that are present in the membrane of cardiac cells. The quantum tunneling of protons results in quantum conductance, which is evaluated to assess its effect on membrane potential. The quantum conductance of extracellular protons is higher than that of intracellular protons. This predicts an inward quantum current of protons through the closed sodium channels. Additionally, the values of quantum conductance are influential and can depolarize the membrane potential according to the quantum version of the GHK equation. The quantum mechanism of depolarization is distinct from other mechanisms because the quantum model suggests that protons can directly depolarize the membrane potential, and not only through indirect effects as proposed by other mechanisms in the literature. Understanding the pathophysiology of arrhythmias mediated by depolarization during acidosis is crucial to treat and control them and to improve the overall clinical outcomes of patients.

## 1. Introduction

In the human body, acid-base balance is under tight regulations because the function of cells requires normal plasma pH levels, and only a minimal disturbance in the blood acidity could affect cells significantly and render them unable to work. Normal blood pH values range between 7.36 to 7.44 and acidemia is defined as extracellular pH less than 7.36. Hence, acidosis is the pathological process that leads to a state of high hydrogen ions (protons) concentration in the plasma (acidemia) if left untreated [1,2].

Acidosis harmfully affects different body systems including the cardiovascular system, nervous system, gastrointestinal system, and others. Here, our focus is the harmful effects of acidosis on the electrical cardiac functions. Acidosis is considered to be an arrhythmogenic factor that predisposes the heart to develop different arrhythmias including ventricular fibrillation, and it can slow the electrical conduction through the atrioventricular node [3,4,5]. Moreover, acidosis depresses the contractile function of the heart and may lead to cardiac arrest and even death in cases of severe acidosis [6]. Depolarization induced by acidosis is a major effect that contributes to the development of cardiac arrhythmias and even cardiac arrest, especially in cases of large and prolonged depolarization at which most of the sodium channels are inactivated [2,4,6,7,8]. The compromise in the contractility of the heart during prolonged depolarization is similar to the absolute refectory period of action potential during which no stimulus can trigger another action potential because sodium channels have been inactivated, and further depolarization will not open these inactivated channels. In addition to that, most of the sodium channels are inactivated and a small percentage will be in the closed state especially when the prolonged depolarization is large, hence this small percentage of channels will not be enough to trigger new action potential when they open in response to a stimulus. This depolarization is present at a resting state, during repolarization phase of action potential (early after-depolarization), and after repolarization (delayed after-depolarization) [4].

The exact mechanism of how acidosis causes depolarization is still not well understood and requires further investigation to fill the knowledge gap [4]. However, different mechanisms are proposed to explain this depolarization [4]: (1) Depolarized resting membrane potential: the first mechanism states that Na^+^/K^+^ ATPase is indirectly inhibited because acidosis inhibits the cellular metabolism and hence production of ATP, but this seems unlikely to happen because first acidosis does not inhibit the metabolism rigorously, and secondly because changes in membrane potential were noticed during times of normal intracellular ATP levels [4]. The second mechanism states that resting intracellular Ca^+2^ concentration is increased, because of this, Na^+^/Ca^+2^ exchanger may be activated or non-specific intracellular cation currents might be generated, but the fact that the currents generated are decreased by the rising intracellular Na^+^ concentrations that happens during acidosis weakens this theory [4]. The third mechanism states that there is a decrease in K^+^ currents during times of acidosis, but this mechanism is disproved somehow because firstly, an increase in intracellular Ca^+2^ during acidosis as mentioned earlier activates calcium activated potassium channels, thus hyper-polarizing the membrane; secondly, increased intracellular sodium concentrations during acidosis might activate sodium activated potassium channels, therefore hyperpolarizing the membrane potential [4]. The fourth mechanism states that potassium piles up in the intercellular clefts of purkinje fibers during acidosis by inhibiting Na^+^/K^+^ ATPase directly, but the gap is that this is difficult to be achieved by merely inhibiting the Na^+^/K^+^ ATPase alone [4]. (2) Early afterdepolarization: acidosis generates early after depolarizations, which are produced by recovery of the inactivated L-type calcium channels during the repolarization phase of action potential, but the gap here is that acidosis inhibits calcium current directly and indirectly by increasing the intracellular calcium concentration [4]. (3) Delayed after depolarization: these are caused by increased intracellular Ca^+2^ which provokes inward depolarizing currents mainly by activating the Na^+^/Ca^+2^ exchanger, and this mechanism is opposed by the fact that the concentration of intracellular sodium ions increases during acidosis and this inhibits the currents mediated by Na^+^/Ca^+2^ exchanger [4].

Accordingly, it seems that these mechanisms are opposed by other mechanisms that counteract the depolarization. Furthermore, none of these mechanisms focus on the protons themselves, which are the direct cause behind the decrease in pH and acidemia. Additionally, the different phases of depolarization cannot be unified into one comprehensive mechanism. Therefore, finding other mechanisms that may underlie the pathophysiology of acidosis-induced depolarization and arrhythmias is warranted to improve the clinical outcomes of acidosis.

The present study aims to propose a possible explanation of the mechanism by which acidosis leads to depolarization by using the quantum tunneling model of ions [9,10,11] and applying it to protons (hydrogen ions). This offers a good opportunity to unveil why acidosis is arrhythmogenic in cardiac tissue from the quantum perspective and improve our understanding of the pathophysiology of acidosis-induced depolarization. Eventually, this will lead to better management and better clinical outcome.

Quantum mechanics is the field of physics that pays attention to atomic and subatomic particles’ behavior. The quantum tunneling is the phenomenon where a wavefunction of a particle can propagate through a potential barrier and that its energy is higher than the energy of the particle. The propagation through the barrier depends exponentially on the barrier’s energy, barrier’s length, particle’s mass, and particle’s energy [12]. This quantum phenomenon has been exploited to explain different biological processes and actions. These actions include point DNA mutations induced by protons quantum tunneling, and activity of enzymes mediated by protons quantum tunneling [13].

In the present study, the model of quantum tunneling of protons through the cardiac voltage-gated sodium channels will be used to show that protons can tunnel through the closed channels and depolarize the membrane potential. The quantum mechanism is different from the previously mentioned mechanisms because the quantum mechanism will focus on the protons themselves as a direct contributor to the depolarization, unlike other mechanisms, which attribute this effect to the indirect effects of acidosis on channels and other ions such as sodium ions, potassium ions, and calcium ions. Other qualities of this quantum model will be discussed later.

Evidence of proton leak through voltage-gated ion channels is established. A study on a patient with severe mixed phenotype who presented with conduction disease and dilated cardiomyopathy demonstrated that a proton leak through mutated Nav1.5 mutation (R219H) is responsible for acidifying the cardiac myocytes as well as the development of arrhythmias and dilated cardiomyopathy [14]. Additionally, mutations in the positively charged residue of segment S4 of voltage-gated sodium channel would result in leakiness of the channel for protons and cations [15]. Another study on cardiomyocytes derived from patient-specific human induced pluripotent stem cells proposes a link between mutations in Nav1.5 channels and the pathogenesis of cardiac arrhythmias and dilated cardiomyopathy through generating proton leak [16]. Additionally, a mutation in voltage-gated sodium channel 1.4 (Nav1.4) in skeletal muscles causes inherited periodic paralysis. Mutation in the S4 voltage sensor in the α subunit of Nav1.4 alters the channel properties and leads to a leak of sodium or protons through the voltage sensor causing depolarization [17]. These currents are called gating pore currents (omega currents) that are not conducted through the usual pathway of conduction but conducted through voltage sensor domains as proposed in the literature [14,15,16,17]. This gives the motivation to apply the quantum model, especially that proton leak does not occur through the usual sodium permeation pathway, and the quantum model can explain the proton leak by quantum tunneling through the closed gate of channels, which is a quantum transport different from the classical permeation through the open channels. Additionally, the direct correlation between proton leak, membrane depolarization and cardiac arrhythmias supports the quantum model because it predicts that quantum tunneling of protons through closed gates can depolarize the membrane potential directly and cause arrhythmias, as will be discussed later in the study.

## 2. The Mathematical Model

The probability of quantum tunneling through a potential barrier is calculated after solving the Schrodinger equation and finding the wave-function of the particle and then applying the Born’s rule to find the probability of finding the particle at a certain position. Therefore, the equation that calculates the tunneling probability is [12]:

(1) $$T_{Q}=e^{\frac{-\sqrt{8m}}{\hbar }\int_{X1}^{X2}\sqrt{U(x)-KE}dx},$$

where *T_Q_* is the tunneling probability, *m* is the mass of the particle (Kg), ℏ is the reduced Planck constant ($1.05\times 10^{-34}$ Js), *U(x)* is the energy of the barrier with respect to the position of particle *x*, *KE* is the kinetic energy of the particle, and *x*2–*x*1 is the region where the energy of barrier is higher than the energy of the particle.

The model of quantum tunneling will be applied on protons and sodium ions while passing through the closed activation or closed inactivation gate of the voltage-gated sodium channels that are present in the membrane of cardiac cells. In the previous works, the full derivation of the tunneling probability equation of ions through the gate was discussed and explained extensively. Therefore, in this study we will use the final form of the derivation [10,11]:

(2) $$T_{Q}=e^{\frac{-\sqrt{8m}}{\hbar }\times \frac{2w}{3E_{gate}}\sqrt{{\left(E_{gate}-KE\right)}^{3}}},$$

where $E_{gate}$ is the energy required for ions to pass through the closed gate (activation or inactivation gate) and its unit is Joule (J), and *w* is the width or the length of the gate and its unit is meter (m).

**Figure 1:**
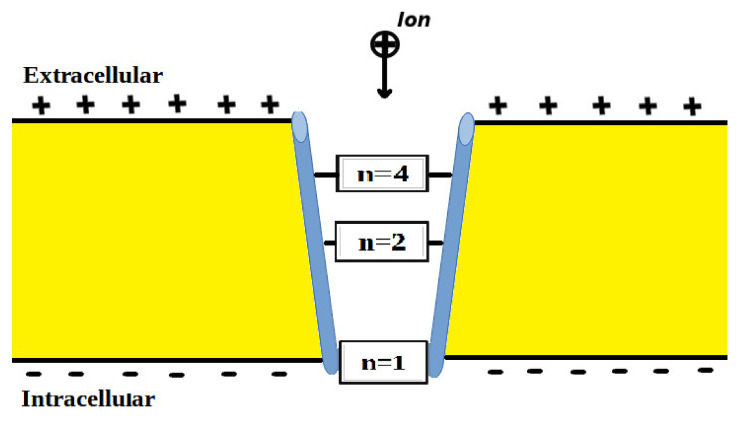
Represents the different locations of the gate through which quantum tunneling of ions occur. n = 1 is where the ion will pass through the entire membrane potential, n = 2 is where the ion will pass the half of membrane potential, and n = 4 is where the ion will pass the quarter of membrane potential.

The voltage-gated channels form an activation gate or inactivation gate at the intracellular end of the membrane. Therefore, extracellular cations that come from outside will pass through the membrane potential (negative inside with respect to outside) to obtain kinetic energy equivalent to $qV_{m}$ and an average thermal energy at body temperature of 310 K equivalent to $\frac{3}{2}K_{B}T=0.64\times 10^{-20}J$ [10]. On the other hand, intracellular cations will have an average thermal energy only [10]. As we mentioned previously, the model is applied on the closed activation and inactivation gates. The activation gate is located at the intracellular end as a hydrophobic constriction from the four S6 segments [18,19]. On the other hand, there are two types of inactivation gate: (1) fast inactivation gate and (2) slow inactivation gate, which have different proposals regarding their locations including the intracellular end and up to the selectivity filter [20,21,22]. Therefore, to account for different locations, we integrate the effect of gate location into the equation of quantum tunneling. As the location moves up from the intracellular end to the extracellular end, the membrane potential available for extracellular cations will be reduced and their kinetic energy will decrease. Accordingly, we choose three different locations: (1) n = 1: this location is at the intracellular end and the ion will pass through the entire membrane potential and the consequent kinetic energy will be $qV_{m}$, (2) n = 2: this location is higher than (1) and the ion will pass through the half of membrane potential and the consequent kinetic energy will be $\frac{qV_{m}}{2}$, and (3) n = 4: this location is higher than (2) and the ion will pass through the quarter of membrane potential and the consequent kinetic energy will be $\frac{qV_{m}}{4}$. See Figure 1. Therefore, we choose these arbitrary values of (n) in a doubling manner to show the influence of the level of the gate on the kinetic energy according to this equation $\frac{qV_{m}}{n}$ and its influence on the quantum tunneling probability in the upcoming sections. However, any value of (n) can be chosen to show its effect on the kinetic energy of ion and on the tunneling probability.

As a result, the tunneling probability for extracellular cations and intracellular cations, respectively, are:

(3) $$T_{Q}(E)=e^{\frac{-\sqrt{8m}}{\hbar }\times \frac{2w}{3E_{gate}}\sqrt{{\left(E_{gate}-(\frac{qV_{m}}{n}+\frac{3}{2}K_{B}T)\right)}^{3}}},$$

(4) $$T_{Q}(I)=e^{\frac{-\sqrt{8m}}{\hbar }\times \frac{2w}{3E_{gate}}\sqrt{{\left(E_{gate}-(\frac{3}{2}K_{B}T)\right)}^{3}}},$$

where *(E)* refers to extracellular ions, *(I)* refers to intracellular ions, *q* is the charge of ion, *V_m_* is the membrane potential, *K_B_* is the Boltzmann constant ($1.38\times 10^{-23}$ J/K), *T* is the body temperature (310 K). Our model will be applied on protons and sodium ions, and both have charge equal to the charge of electron $1.6\times 10^{-19}C$. The mass of proton is $1.67\times 10^{-27}$ kg and the mass of sodium ion is $3.8\times 10^{-26}$ kg.

The conductance of single channel is an important property of the channel that determines the electrical features of excitable tissues. In the context of the quantum model, we will deal with quantum conductance of a single channel $C_{Q}$ [10,19]:

(5) $$C_{Q}=\frac{q^{2}}{h}T_{Q},$$

where *q* is charge of the ion, *h* is the Planck constant ($6.6\times 10^{-34}$ Js), and *T_Q_* is the tunneling probability. The unit of quantum conductance of single channel is Siemens (S).

Eventually, we will consider the quantum membrane conductance $MC_{Q}$ to evaluate the influence of quantum tunneling on the membrane potential [8,10]:

(6) $$MC_{Q}=D\times C_{Q},$$

where *D* is the density of channels (channels/cm^2^), and $C_{Q}$ is the quantum conductance of a single channel with the unit of (mS). Thus, the unit of quantum membrane conductance is mS/cm^2^.

The membrane conductance due to open channels MC is [8]:

(7) MC=D×C,

Here, the difference is that the conductance of single channel (when it is open) *C* is constant, however, the quantum conductance of single channel $C_{Q}$ depends on the quantum tunneling probability through the closed gate.

To assess the impact of quantum tunneling of protons and sodium ions on the resting membrane potential, the quantum version of Goldman–Hodgkin–Katz (GHK) equation will be used [8,10]:

(8) $${\left[K\right]}_{E}(MC_{K})+{\left[Na\right]}_{E}(MC_{Na}+MC_{Q}{\left(Na\right)}_{E})+{\left[H\right]}_{E}(MC_{Q}{\left(H\right)}_{E})=e^{\frac{FV_{m}}{RT}}({\left[K\right]}_{I}(MC_{K})+{\left[Na\right]}_{I}(MC_{Na}+MC_{Q}{\left(Na\right)}_{E})+{\left[H\right]}_{I}(MC_{Q}{\left(H\right)}_{E})),$$

where [ ] refers to the concentration, *(E)* indicates extracellular ions, *(I)* indicates intracellular ions, *(K)* refers to potassium ions, *(Na)* refers to sodium ions, (*H*) refers to protons (hydrogen ions), $MC_{Na}$ is the resting membrane conductance of sodium ions due to leaky channels, $MC_{Q}{\left(Na\right)}_{E}$ is the quantum membrane conductance of extracellular sodium ions, $MC_{Q}{\left(Na\right)}_{I}$ is the quantum membrane conductance of intracellular sodium ions, $MC_{K}$ is the resting membrane conductance of potassium ions due to leaky channels, $MC_{Q}{\left(H\right)}_{E}$ is the quantum membrane conductance of extracellular protons, $MC_{Q}{\left(H\right)}_{I}$ is the quantum membrane conductance of intracellular protons, *F* is Faraday’s constant (96,485.33 C/mol), *R* is the gas constant (8.31 J/Kmol), *T* is body temperature (310 K), and *V_m_* is resting membrane potential.

In the present paper, quantum conductance of protons and sodium will be studied for the purpose of comparison.

Before considering the quantum conductance of protons and sodium ions, the resting membrane potential $V_{m}=0.087$ V [2,8] (negative inside with regard to outside) if the following physiological parameters are substituted in Equation (8): ${\left[Na\right]}_{E}=142$ mEq/L [2,8], ${\left[Na\right]}_{I}=14$ mEq/L [2,8], ${\left[K\right]}_{E}=4$ mEq/L [2,8], ${\left[K\right]}_{I}=140$ mEq/L [2,8], $MC_{Na}=0.005$ mS/cm^2^ [2,8], and $MC_{K}=0.5$ mS/cm^2^ [2,8].

## 3. Results

In this section, a mathematical evaluation of quantum tunneling probability, quantum conductance of single channel, and quantum membrane conductance is considered. This evaluation is based on graphing the relationships between the quantum variables and the energy of gate under the influence of different factors.

By considering Equation (3), the tunneling probability of extracellular protons is calculated by the following equation:

(9) $$T_{Q}{\left(H\right)}_{E}=e^{\frac{-7.35L}{E_{Gate}}\sqrt{{\left(E_{Gate}-\frac{16V_{m}}{n}-0.64\right)}^{3}}},$$

On the other hand, the tunneling of probability of intracellular protons is calculated by the following equation considering Equation (4):

(10) $$T_{Q}{\left(H\right)}_{I}=e^{\frac{-7.35L}{E_{Gate}}\sqrt{{\left(E_{Gate}-0.64\right)}^{3}}},$$

From Equation (2), we took $10^{-20}$ as a common factor from the square root so that the charge of ion becomes 16, thermal kinetic energy 0.64, and $E_{Gate}=\frac{E_{gate}}{10^{-20}}$ as in Equations (9) and (10). Additionally, the number −7.35 emerges when we substitute the constants in Equation (2) taking into consideration that $L=\frac{w}{10^{-10}}$ (and multiplying the exponent by $10^{-10}$), $E_{Gate}=\frac{E_{gate}}{10^{-20}}$ (and dividing the exponent by $10^{-20}$), and multiplying by $\sqrt{{\left(10^{-20}\right)}^{3}}=10^{-30}$ which is the common factor taken from the square root. This is made to simplify the equations and to make it easy to deal with numbers. So, the number 7.35 is a result of the following calculations: $\frac{\sqrt{8\times 1.67\times 10^{-27}}}{1.05\times 10^{-34}}\times \frac{2\times 10^{-10}\times 10^{-30}}{3\times 10^{-20}}=7.35$.

In this section, the focus is on the energy of gate $E_{Gate}$ (either the activation or inactivation gate) which represents the energy barrier of ions passage when the gates are closed. We focus on the energy of the gate because acidosis increases the energy required to inactivate channels by shifting the half inactivation voltage to the right [23]. Therefore, the structure of the inactivated channel is destabilized and the energy barrier of ions passage through the closed destabilized gate decreases. On the other hand, acidosis increases the energy required to open the closed activation gate by shifting the half activation voltage to the right [23]. Therefore, the energy barrier of ions passage through the closed activation gate increases. However, the pathological processes associated with acidosis such as hypoxia, ischemia, and inflammation increase the energy of gate $E_{Gate}$ for inactivation gate and decrease the energy of gate $E_{Gate}$ for the activation gate by shifting the half activation voltages to the left [24]. Thus, it seems that acidosis and their related pathologies have opposite actions on the gates, however, it is difficult to determine the final outcome. Hence, the overall decrease in the gate’s energy $E_{Gate}$ is a likely and possible outcome for both types of gates, or at least one of them.

The gating charge of Nav1.5 channels is $q_{gating}=3.8e$ [23] and the half activation voltage $V_{1/2}=0.0326$ V [23]. Therefore, we can estimate the energy of the gate using this equation $q_{gating}(V_{1/2}-V_{m})$ where *V_m_* is chosen to be the resting membrane potential $V_{m}=0.087$ V which represents the original and initial state of potential. As a result, $E_{Gate}=3.31$ J and can be 4.35 J if the gating charge is 5e [25]. On the other hand, this estimation cannot be used for the inactivation gate because the increase in half inactivation voltage should cause the energy of the gate to decrease since more energy is needed to inactivate the channel in this case. Thus, using this estimation will increase the energy of the inactivation gate instead of decreasing it. In other words, this equation $q_{gating}(V_{1/2}-V_{m})$ can estimate the energy to inactivate the channel but not the gate’s energy $E_{Gate}$ that impedes the ions passage. Because these values are based on estimation, we will take a range of values for $E_{Gate}$ to include a wide range of possibilities for activation and inactivation gates. Additionally, the ranges will be chosen so that the substitution will not result in negative numbers in the square root in the equations of quantum tunneling (avoiding obtaining imaginary numbers). In our study, we assume that the energy of gate $E_{Gate}$ for both activation and inactivation gates is the same to simplify the mathematical evaluation of the effect of $E_{Gate}$ on quantum tunneling probability, quantum conductance of single channel, and quantum membrane conductance.

In the following graphs, these setting values will be substituted in the equations unless we set different values for evaluation: L=1.5 m [10], $V_{m}=0.087$ V [10], n=1 [10], and $D=10^{11}$ channels/cm^2^ [8].

**Figure 2:**
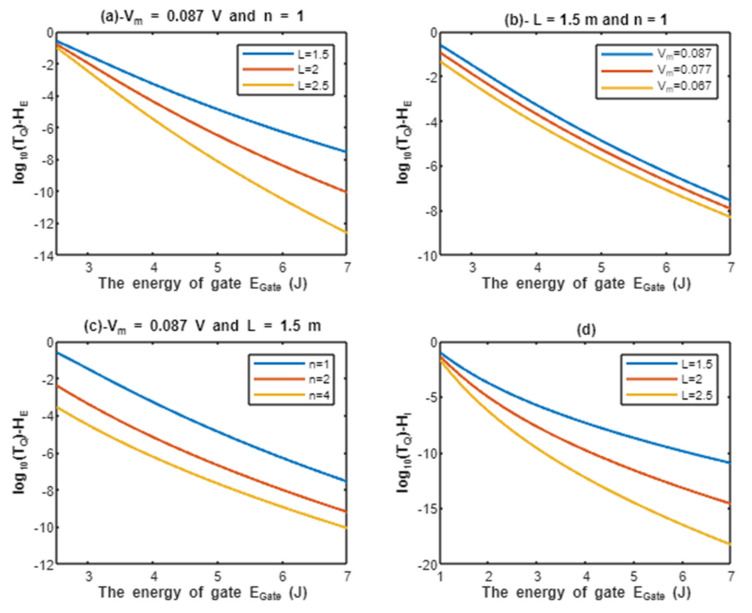
(**a**–**c**): represents the mathematical graph of common logarithm of tunneling probability for extracellular protons $\log_{10}(T_{Q})-H_{E}$ over gate’s energy range from 2.5 to 7 J according to gate length, membrane potential, and gate location, respectively; (**d**): represents the mathematical graph of common logarithm of tunneling probability for intracellular protons $\log_{10}(T_{Q})-H_{I}$ over gate’s energy range from 1 to 7 J according to gate length.

According to Equations (9) and (10), the common logarithms of tunneling probability of extracellular protons $\log_{10}(T_{Q})-H_{E}$ and intracellular protons $\log_{10}(T_{Q})-H_{I}$ are evaluated with regard to the energy of gate $E_{Gate}$ under the influence of different factors. See Figure 2.

Above each graph in the previous figure, we put setting values at which the evaluation of the relationship is based on. For example, in graph (a) of Figure 2 the plotting of the relationship between the common logarithm of tunneling probability of extracellular protons and the energy of gate is made by substituting the above setting values $V_{m}=0.087$ V and n = 1 in Equation (9) and by making the energy of the gate as the variable on the X-axis and the common logarithm of tunneling probability as the variable on the Y-axis. The relationship is plotted three times by substituting the setting values and each time, we substitute different value of gate length *L* (*L* = 1.5 m, *L* = 2 m, and *L* = 2.5 m) in Equation (9) to produce three plots. Each graph has its own setting values according to the factor that the graph, which is plotted three times, is based on. This style of evaluation will be applied to all graphs of this study. Additionally, this style of evaluation is used to facilitate the understanding of the influence of gate length, membrane potential, gate location, and the channels density on the tunneling probability and quantum conductance. Additionally, this style of evaluation of setting certain reasonable values of the variables helps to obtain consequent numerical values that can aid in the understanding of the behavior of the relationship and can be used for the purposes of comparison for different ions at the same setting values. Moreover, graph (d) of Figure 2 does not contain setting values because intracellular ions do not depend on membrane potential or the location of gate. Therefore, no setting values are required to assess the relationship.

By considering Equation (3), the tunneling probability of extracellular sodium ions is calculated by the following equation:

(11) $$T_{Q}{\left(Na\right)}_{E}=e^{\frac{-35L}{E_{Gate}}\sqrt{{\left(E_{Gate}-\frac{16V_{m}}{n}-0.64\right)}^{3}}},$$

By considering Equation (4), the tunneling probability of intracellular sodium ions is calculated by the following equation:

(12) $$T_{Q}{\left(Na\right)}_{I}=e^{\frac{-35L}{E_{Gate}}\sqrt{{\left(E_{Gate}-0.64\right)}^{3}}},$$

The same mathematical manipulations made on the equation of tunneling probability of protons are made on the equation of tunneling probability of sodium ions. The number 35 emerges as a result of the following calculations: $\frac{\sqrt{8\times 3.8\times 10^{-26}}}{1.05\times 10^{-34}}\times \frac{2\times 10^{-10}\times 10^{-30}}{3\times 10^{-20}}=35$.

**Figure 3:**
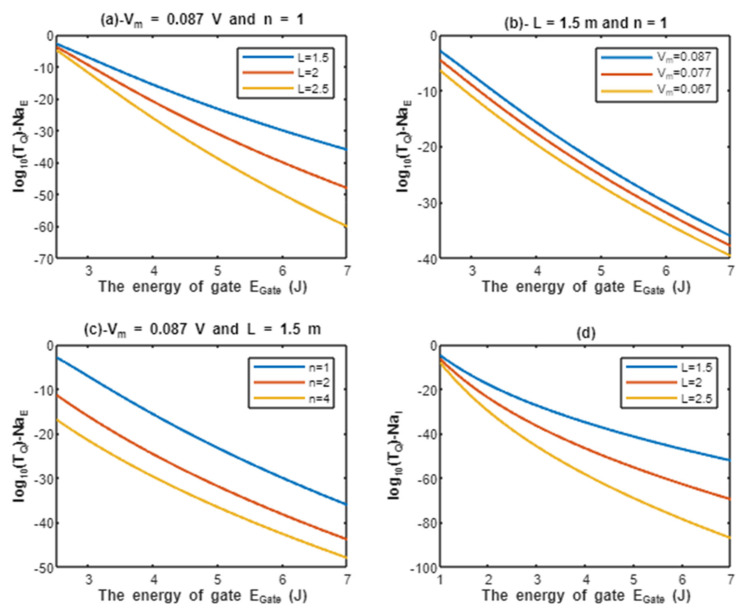
(**a**–**c**): represents the mathematical graph of common logarithm of tunneling probability for extracellular sodium ions $\log_{10}(T_{Q})-Na_{E}$ over gate’s energy range from 2.5 to 7 J according to gate length, membrane potential, and gate location, respectively; (**d**): represents the mathematical graph of common logarithm of tunneling probability for intracellular sodium ions $\log_{10}(T_{Q})-Na_{I}$ over gate’s energy range from 1 to 7 J according to gate length.

According to Equations (11) and (12), the common logarithms of tunneling probability of extracellular sodium ions $\log_{10}(T_{Q})-Na_{E}$ and intracellular sodium ions $\log_{10}(T_{Q})-Na_{I}$ are evaluated with regard to the energy of gate $E_{Gate}$ under the influence of different factors. See Figure 3.

By considering Equation (5), the quantum conductance of a single channel for extracellular protons is calculated by the following equation:

(13) $$C_{Q}{\left(H\right)}_{E}=3.88\times 10^{-5}e^{\frac{-7.35L}{E_{Gate}}\sqrt{{\left(E_{Gate}-\frac{16V_{m}}{n}-0.64\right)}^{3}}},$$

On the other hand, the quantum conductance of a single channel for intracellular protons is calculated by the following equation considering Equation (5):

(14) $$C_{Q}{\left(H\right)}_{I}=3.88\times 10^{-5}e^{\frac{-7.35L}{E_{Gate}}\sqrt{{\left(E_{Gate}-0.64\right)}^{3}}},$$

The unit of quantum conductance of a single channel is (S).

The constant $3.88\times 10^{-5}$ emerges after substituting the values of constants in Equation (5). So, $\frac{q^{2}}{h}=\frac{{\left(1.6\times 10^{-19}\right)}^{2}}{6.6\times 10^{-34}}=3.88\times 10^{-5}$.

**Figure 4:**
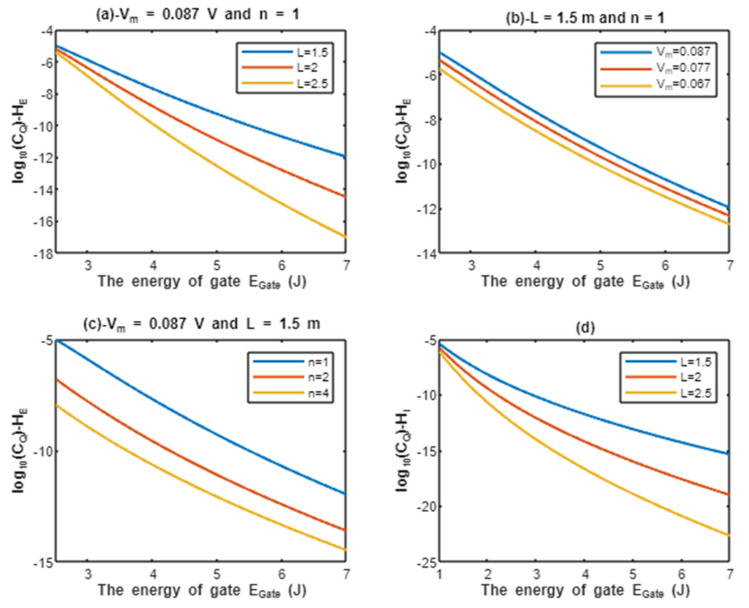
(**a**–**c**): represents the mathematical graph of common logarithm of quantum conductance of single channel for extracellular protons $\log_{10}(C_{Q})-H_{E}$ over gate’s energy range from 2.5 to 7 J according to gate length, membrane potential, and gate location, respectively; (**d**): represents the mathematical graph of common logarithm of quantum conductance of single channel for intracellular protons $\log_{10}(C_{Q})-H_{I}$ over gate’s energy range from 1 to 7 J according to gate length.

According to Equations (13) and (14), the relationship between the common logarithms of quantum conductance of a single channel for extracellular protons $\log_{10}(C_{Q})-H_{E}$ and intracellular protons $\log_{10}(C_{Q})-H_{I}$, and the energy of gate $E_{Gate}$ is evaluated under the influence of different factors. See Figure 4.

By considering Equation (5), the quantum conductance of single channel for extracellular sodium ions:

(15) $$C_{Q}{\left(Na\right)}_{E}=3.88\times 10^{-5}e^{\frac{-35L}{E_{Gate}}\sqrt{{\left(E_{Gate}-\frac{16V_{m}}{n}-0.64\right)}^{3}}},$$

On the other hand, the quantum conductance of single channel for intracellular sodium ions is calculated by the following equation considering Equation (5):

(16) $$C_{Q}{\left(Na\right)}_{I}=3.88\times 10^{-5}e^{\frac{-35L}{E_{Gate}}\sqrt{{\left(E_{Gate}-0.64\right)}^{3}}},$$

The unit of quantum conductance of a single channel is (S).

The constant $3.88\times 10^{-5}$ emerges after substituting the values of constants in Equation (5). So, $\frac{q^{2}}{h}=\frac{{\left(1.6\times 10^{-19}\right)}^{2}}{6.6\times 10^{-34}}=3.88\times 10^{-5}$.

**Figure 5:**
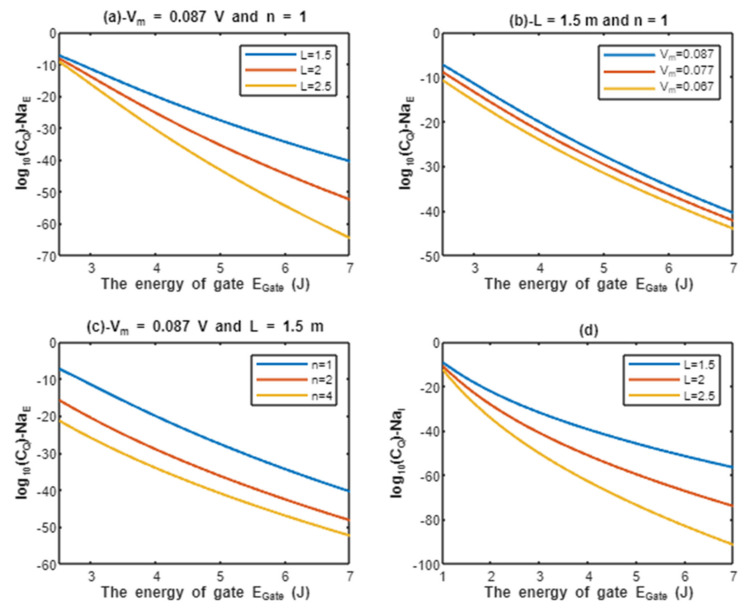
(**a**–**c**): represents the mathematical graph of common logarithm of quantum conductance of single channel for extracellular sodium ions $\log_{10}(C_{Q})-Na_{E}$ over gate’s energy range from 2.5 to 7 J according to gate length, membrane potential, and gate location, respectively; (**d**): represents the mathematical graph of common logarithm of quantum conductance of single channel for intracellular sodium ions $\log_{10}(C_{Q})-Na_{I}$ over gate’s energy range from 1 to 7 J according to gate length.

According to Equations (15) and (16), the relationship between the common logarithms of quantum conductance of single channel for extracellular sodium ions $\log_{10}(C_{Q})-Na_{E}$ and intracellular sodium ions $\log_{10}(C_{Q})-Na_{I}$, and the energy of gate $E_{Gate}$ is evaluated according to different factors. See Figure 5.

By considering Equation (6),the quantum membrane conductance of extracellular protons can be calculated by the following equation:

(17) $$MC_{Q}{\left(H\right)}_{E}=3.88\times 10^{-2}\times D\times e^{\frac{-7.35L}{E_{Gate}}\sqrt{{\left(E_{Gate}-\frac{16V_{m}}{n}-0.64\right)}^{3}}},$$

On the other hand, the quantum membrane conductance of intracellular protons can be calculated by the following equation considering Equation (6):

(18) $$MC_{Q}{\left(H\right)}_{I}=3.88\times 10^{-2}\times D\times e^{\frac{-7.35L}{E_{Gate}}\sqrt{{\left(E_{Gate}-0.64\right)}^{3}}},$$

The unit of quantum membrane conductance is mS/cm^2^.

$3.88\times 10^{-5}$ is converted to $3.88\times 10^{-2}$ by multiplying by $10^{3}$ to convert the unit of conductance from (S) to (mS) so that the unit of quantum membrane conductance is mS/cm^2^.

**Figure 6:**
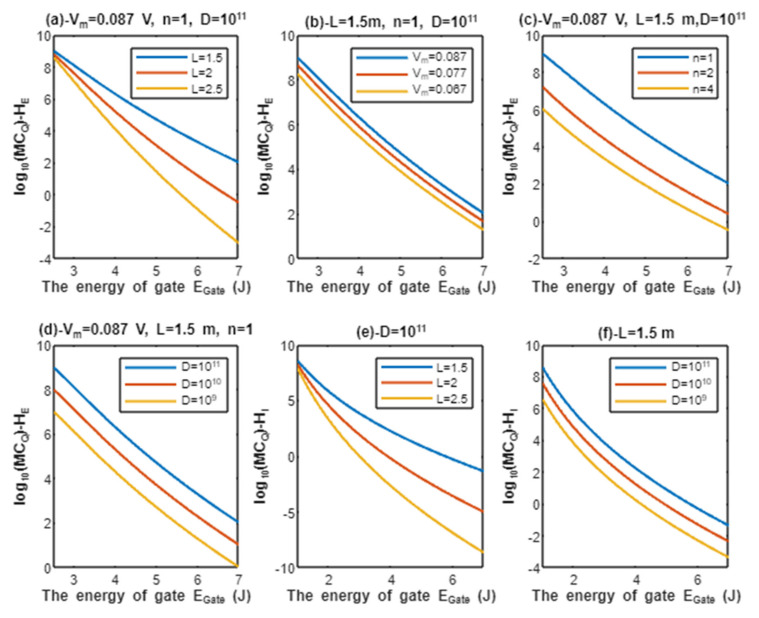
(**a**–**d**): represents the mathematical graph of common logarithm of quantum membrane conductance for extracellular protons $\log_{10}(MC_{Q})-H_{E}$ over gate’s energy range from 2.5 to 7 J according to gate length, membrane potential, gate location, and channels density, respectively; (**e**,**f**): represents the mathematical graph of common logarithm of quantum membrane conductance for intracellular protons $\log_{10}(MC_{Q})-H_{I}$ over gate’s energy range from 1 to 7 J according to gate length, and channels density, respectively.

According to Equations (17) and (18), the relationship between the common logarithms of quantum membrane conductance of extracellular protons $\log_{10}(MC_{Q})-H_{E}$ and intracellular protons $\log_{10}(MC_{Q})-H_{I}$, and the energy of gate $E_{Gate}$ is evaluated according to different factors. See Figure 6.

By considering Equation (6), the quantum membrane conductance of extracellular sodium ions can be calculated by the following equation:

(19) $$MC_{Q}{\left(Na\right)}_{E}=3.88\times 10^{-2}\times D\times e^{\frac{-35L}{E_{Gate}}\sqrt{{\left(E_{Gate}-\frac{16V_{m}}{n}-0.64\right)}^{3}}},$$

By considering Equation (6), the quantum membrane conductance of intracellular sodium ions can be calculated by the following equation:

(20) $$MC_{Q}{\left(Na\right)}_{I}=3.88\times 10^{-2}\times D\times e^{\frac{-35L}{E_{Gate}}\sqrt{{\left(E_{Gate}-0.64\right)}^{3}}},$$

The unit of quantum membrane conductance is mS/cm^2^.

$3.88\times 10^{-5}$ is converted to $3.88\times 10^{-2}$ by multiplying by $10^{3}$ to convert the unit of conductance from (S) to (mS) so that the unit of quantum membrane conductance is mS/cm^2^.

**Figure 7:**
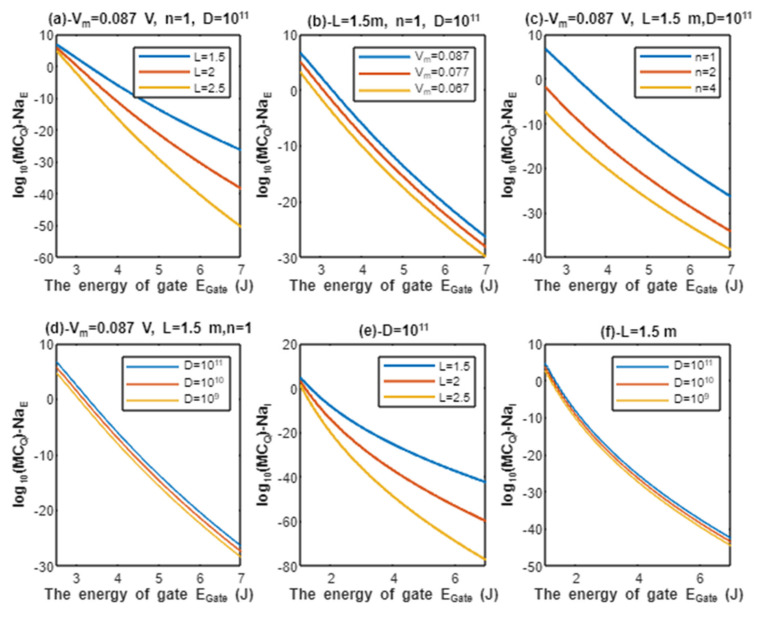
(**a**–**d**): represents the mathematical graph of common logarithm of quantum membrane conductance for extracellular sodium ions $\log_{10}(MC_{Q})-Na_{E}$ over gate’s energy range from 2.5 to 7 J according to gate length, membrane potential, gate location, and channels density, respectively; (**e**,**f**): represents the mathematical graph of common logarithm of quantum membrane conductance for intracellular sodium ions $\log_{10}(MC_{Q})-Na_{I}$ over gate’s energy range from 1 to 7 J according to gate length and channels density, respectively.

According to Equations (19) and (20), the relationship between the common logarithms of quantum membrane conductance of extracellular sodium ions $\log_{10}(MC_{Q})-Na_{E}$ and intracellular sodium ions $\log_{10}(MC_{Q})-Na_{I}$, and the energy of gate $E_{Gate}$ is evaluated under the influence of different factors. See Figure 7.

Here, the quantum version of GHK equation, as in Equation (8), is used to assess the impact of quantum tunneling of protons on the resting membrane potential without considering the quantum tunneling of sodium ions:

(21) $$2.71+10^{-pH_{E}+3}\times 3.88\times 10^{-2}\times D\times e^{\frac{-7.35L}{E_{Gate}}\sqrt{{\left(E_{Gate}-\frac{16V_{m}}{n}-0.64\right)}^{3}}}=e^{-37.45V_{m}}(70.07+10^{-pH_{E}+4}\times 3.88\times 10^{-2}\times D\times e^{\frac{-7.35L}{E_{Gate}}\sqrt{{\left(E_{Gate}-0.64\right)}^{3}}}),$$

where $10^{-pH_{E}+3}$ is the extracellular concentration of protons in mEq/L and $10^{-pH_{E}+4}$ is the intracellular concentration of protons in mEq/L. We assume that the intracellular concentration of protons is higher than the extracellular concentration by one unit of pH, hence the ‘+4’ instead of ‘+3’. This is because the intracellular pH ranges between 6–7.4 [2] and the intracellular pH also falls down during the process of acidosis [2,26]. The $pH_{E}$ is 7.4 and the $pH_{I}$ is 6.4 (the difference is one unit) and the ratio between the intracellular concentration to the extracellular concentration is 10. These values are substituted unless we set different values for evaluation.

The value 2.71 results from the following calculations by substituting the values of physiological parameters in Equation (8): $\left(142\times 0.005\right)+(4\times 0.5)=2.71$, the value of 70.07 results from the following calculations: (14×0.005)+(140×0.5)=70.07, and the value 37.45 results from the following calculations: $\frac{96485.33}{8.31\times 310}=37.45$.

The minus sign is inserted in the mathematical term $e^{-37.45V_{m}}$ because the absolute value of membrane potential is desired since $V_{m}$ in this term $16V_{m}$ must be substituted as an absolute value to get positive value of kinetic energy for extracellular cations. In Equation (21), we assume that the concentrations of sodium ions and potassium ions are constant during the process of acidosis.

**Figure 8:**
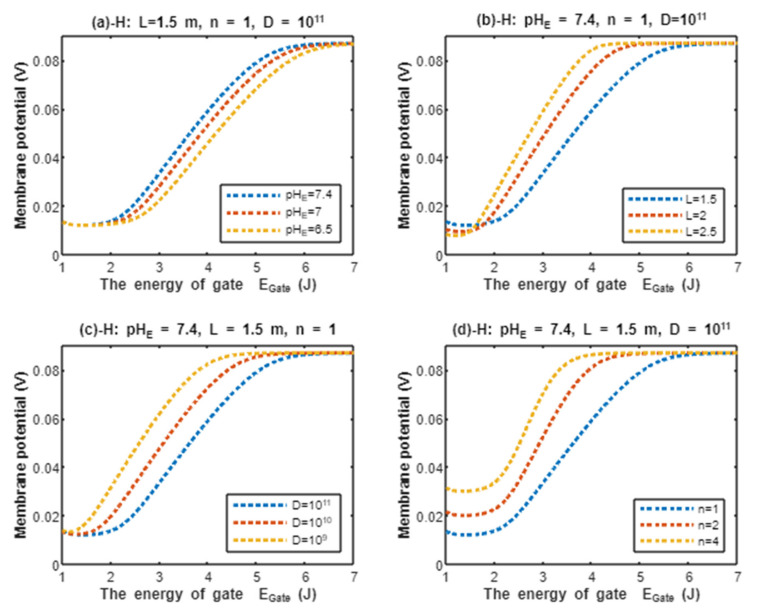
The relationship between the resting membrane potential and the energy of gate under the influence of quantum tunneling of protons according to external pH, gate length, channels density, and gate location.

According to Equation (21), the resting membrane potential is assessed with regard to the energy of gate $E_{Gate}$ under the influence of quantum tunneling of protons and according to different factors. See Figure 8.

For the purpose of comparison, the quantum version of GHK equation, as in Equation (8), is used to assess the effect of quantum tunneling of sodium ions on the resting membrane potential without considering the quantum tunneling of protons:

(22) $$2.71+5.51\times D\times e^{\frac{-35L}{E_{Gate}}\sqrt{{\left(E_{Gate}-\frac{16V_{m}}{n}-0.64\right)}^{3}}}=e^{-37.45V_{m}}(70.07+0.543\times D\times e^{\frac{-35L}{E_{Gate}}\sqrt{{\left(E_{Gate}-0.64\right)}^{3}}}),$$

Here, 5.51 results from the following calculation: $142\times 3.88\times 10^{-2}=5.51$ and 0.543 results from the following calculation: $14\times 3.88\times 10^{-2}=0.543$.

**Figure 9:**
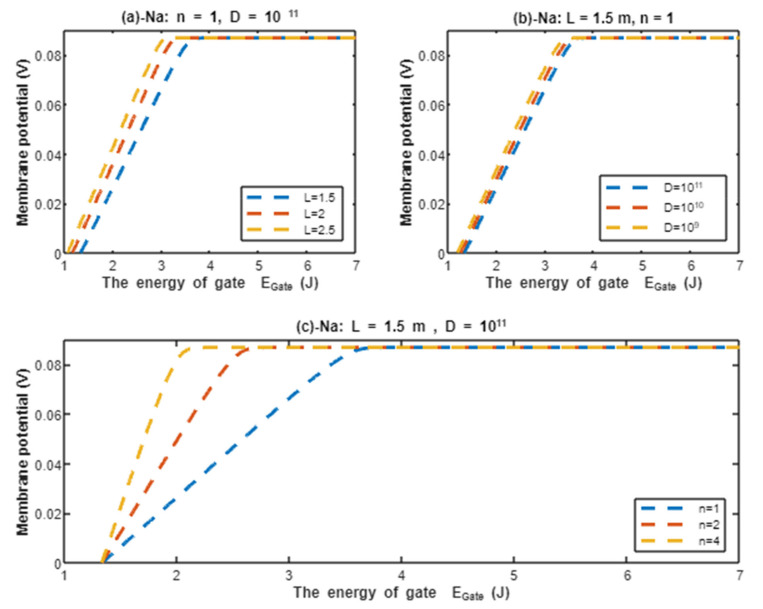
The relationship between the resting membrane potential and the energy of gate under the influence of quantum tunneling of sodium ions according to gate length, channel density, and gate location.

According to Equation (22), the resting membrane potential is assessed with regard to the energy of gate $E_{Gate}$ under the effect of quantum tunneling of sodium ions and according to different factors. See Figure 9.

On the other hand, the classical version of GHK equation is used to evaluate the effect of the transport of protons through open channels. The classical version of GHK equation does not include the quantum conductance of protons or sodium ions:

(23) $${\left[Na\right]}_{E}(MC_{Na})+{\left[K\right]}_{E}(MC_{K})+{\left[H\right]}_{E}(MC_{H})=e^{-37.45V_{m}}({\left[Na\right]}_{I}(MC_{Na})+{\left[K\right]}_{I}(MC_{K})+{\left[H\right]}_{I}(MC_{H})),$$

By substituting the values of physiological parameters in Equation (23):

(24) $$2.71+10^{-pH_{E}+3}\times \frac{P_{H}}{P_{Na}}C_{Na}\times D=e^{-37.45V_{m}}(70.07+10^{-pH_{E}+4}\times \frac{P_{H}}{P_{Na}}C_{Na}\times D),$$

where $C_{Na}$ is the single channel conductance of cardiac sodium channel when the channel is open $17.3\times 10^{-12}$ S [8,27]. Moreover, the $\frac{P_{H}}{P_{Na}}$ is the permeability ratio between protons and sodium ions, which is substituted 252 [28].

(25) $$2.71+10^{-pH_{E}+3}\times 4.36\times 10^{-6}\times D=e^{-37.45V_{m}}(70.07+10^{-pH_{E}+4}\times 4.36\times 10^{-6}\times D),$$

The value $4.36\times 10^{-6}$ is the result of multiplying $17.3\times 10^{-12}$ by 252 and $10^{3}$. The factor $10^{3}$ is used to convert the unit of (S) to (mS).

**Figure 10:**
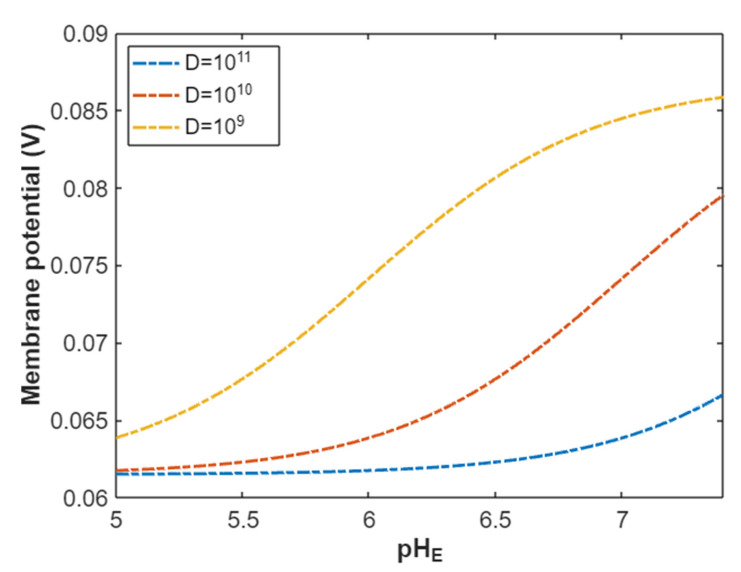
The relationship between the resting membrane potential and a range of external pH from 5 to 7.4 under the influence of classical transport of protons through open voltage-gated sodium channels and at different channels densities.

According to Equation (25), the resting membrane potential can be assessed under the influence of classical transport of protons through open sodium channels. See Figure 10.

## 4. Discussion

The present work proposes a quantum model to explain the acidosis-induced depolarization and the consequent cardiac arrhythmias. The model states that protons are able to pass through the closed voltage-gated sodium channels via quantum tunneling. According to the quantum model, quantum tunneling of protons results in a quantum current that passes through the channel and hence there will be a quantum conductance of single channel and quantum membrane conductance. Therefore, quantum tunneling is the cardinal feature that provides the outcome of quantum conductance. The model is applied on the cardiac sodium channels Nav1.5 specifically at the activation and inactivation gate. Both activation and inactivation gates possess an energy barrier to prevent the passage of ions when these gates are closed. During acidosis, the energy barriers of the gates are affected. It was found that there is a right shift in the activation and inactivation curve under the effect of acidosis and this means that the half activation and half inactivation voltages increase [23]. When the half activation voltage increases, this means that higher energy is needed to open the closed activation gate and the energy barrier for ions passage increases. Moreover, when the half inactivation voltage increases, this means higher energy is needed to close the inactivation gate and the energy barrier for ions passage decreases. On the other hand, acidosis is not a sole pathological entity but it happens as a consequence of other pathological events such as hypoxia, ischemia, infarction, inflammation, and metabolic dysfunction [26]. These events such as ischemia and inflammation cause a paradoxical effect on the half activation and inactivation voltages if they are compared with the effect of acidosis on these voltages. These pathological events cause a left shift in the activation and inactivation curves [24]. Therefore, the final outcome may depend on which mechanisms predominate over other mechanisms and the severity of the mechanisms. However, the overall decrease in the energy barrier for ions passage through the activation or inactivation gates, or even both, is possible and can occur especially that voltage-gated channels become leaky under the effect of these pathological entities [24]. Accordingly, we express the energy barrier by the term ‘the energy of gate $E_{Gate}$’ that represents the energy required for ions to pass through the closed activation or inactivation gate.

**Figure 11:**
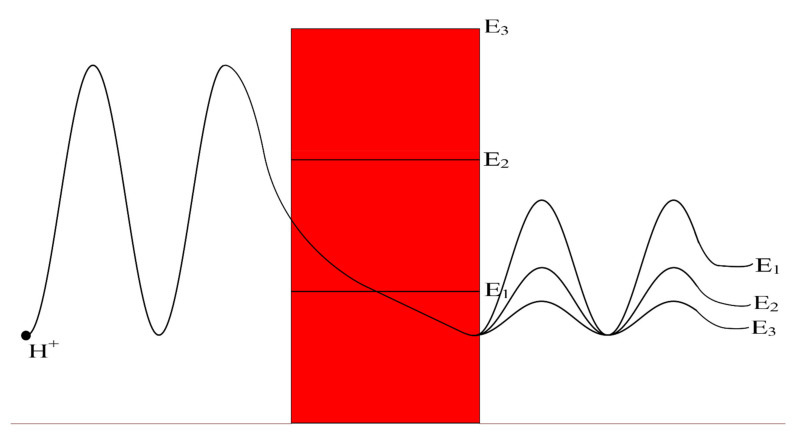
A schematic diagram that represents the quantum tunneling of the wave-function of a proton through different levels of gate energy E3 > E2 > E1. The lower is the gate energy; the higher is the tunneling probability, which is represented by higher amplitude of wave-function after tunneling through the gate (shown in red).

We investigate, mathematically, the quantum tunneling probability and quantum conductance of protons and sodium ions over a range of $E_{Gate}$ because it is the parameter that is influenced during acidosis and its associated pathological processes. Moreover, this mathematical investigation is set under different factors that affect the quantum tunneling including the length of gate, the location of gate, the membrane potential, and the channels density. As the energy of gate decreases, the quantum tunneling probability and the quantum conductance increase as presented in the results section. See Figure 11.

**Figure 12:**
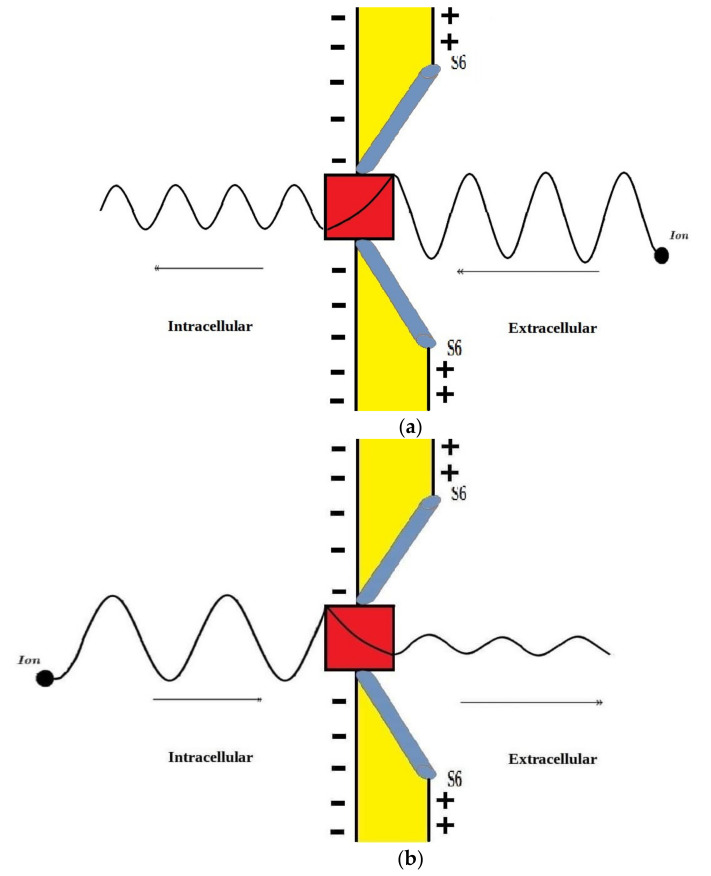
A schematic diagram that represents the quantum tunneling of the wavefunction of extracellular and intracellular ions through the gate (red in color). (**a**): extracellular ion has higher kinetic energy manifested as shorter wavelength, and higher tunneling probability manifested as higher amplitude after passing the gate; (**b**): intracellular ion has lower kinetic energy manifested as longer wavelength and lower tunneling probability manifested as lower amplitude after passing the gate.

According to the graphs of quantum tunneling in Figure 2 and Figure 3, it is clear that extracellular cations (either protons or sodium ions) have higher tunneling probability if it is compared with quantum tunneling of intracellular cations because extracellular cations acquire higher kinetic energy due to their passage through the membrane potential *V_m_*. See Figure 12.

**Figure 13:**
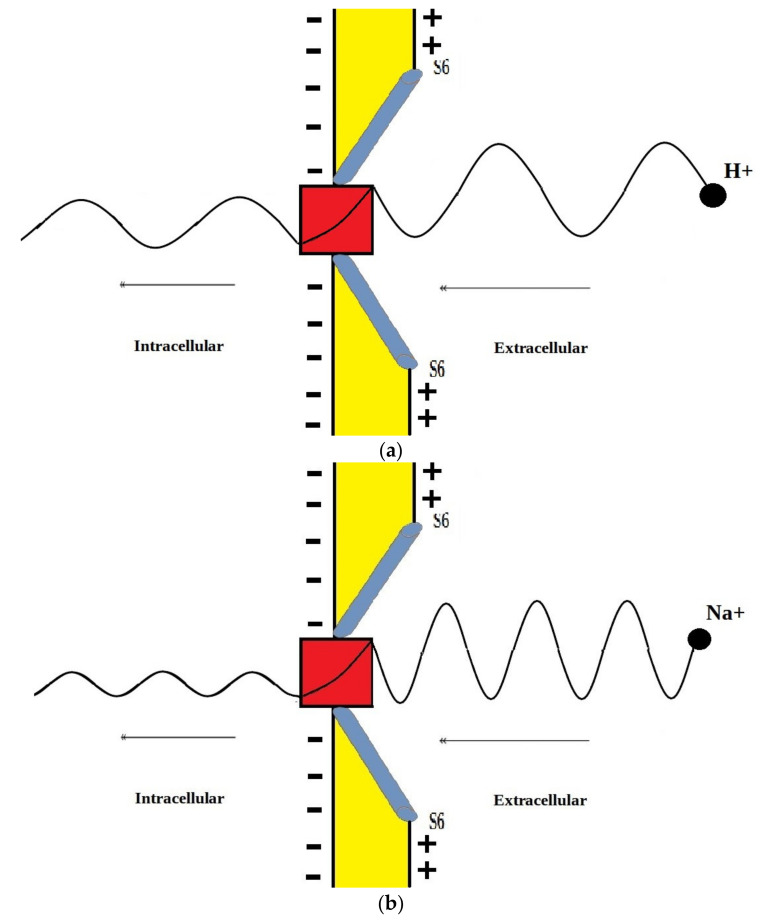
A schematic diagram that represents the quantum tunneling of extracellular proton and sodium ion. (**a**): proton has longer wavelength (due to small mass) and higher tunneling probability manifested as higher amplitude after passing the gate; (**b**): sodium ion has shorter wavelength (due to larger mass) and lower tunneling probability manifested as lower amplitude after passing the gate.

This discrepancy in tunneling probabilities between extracellular and intracellular ions results in a quantum gradient that favors the flow from the extracellular compartment to the intracellular compartment. Additionally, it seems that the discrepancy in tunneling probabilities for protons is less than the discrepancy for sodium ions, however, protons have higher tunneling probabilities than sodium ions because a proton’s mass is less than a sodium ion’s mass. See Figure 13.

Additionally, the length of the gate is an important factor that inversely affects the tunneling probabilities, hence as the length increases, the tunneling probability decreases as presented in the graphs (a) and (d) of Figure 2 and Figure 3. Additionally, the membrane potential (negative inside regarding to outside) affects the tunneling probability of extracellular ions, not intracellular ions. As the absolute value of membrane potential increases, the kinetic energy of extracellular ions and their tunneling probability increase as presented in the graph (b) of Figure 2 and Figure 3. The protons tunneling occurs through the activation gate and inactivation gate and the location of these gates is a crucial factor that affects the tunneling probability. The activation gate is located at the intracellular end (n =1) which guarantees extracellular ions to pass almost through the entire membrane potential to acquire higher kinetic energy. The fast and slow inactivation gates can be located at the intracellular end (n = 1), however, they can also be located away from the intracellular end, reducing the membrane potential available for extracellular ions to obtain kinetic energy. To include wide possibilities for the locations of the gate, we investigate the effect of different levels of the gate’s location (n = 1, n = 2, and n = 4) on the tunneling probability. Each level reduces the membrane potential by 1/n. This means as (n) increases, the tunneling probability of extracellular ions decreases as presented in the graph (c) of Figure 2 and Figure 3. A numerical comparison between protons and sodium ions in terms of quantum tunneling probability according to the different factors will be useful to make the results clear and comprehensible and to make it easier to notice the differences between the extracellular and the intracellular ions and the differences between protons and sodium ions according to the different setting values. The graphs in the results section evaluate the quantum tunneling probability using the common logarithm $\log_{10}$, but in the following numerical description, the values of tunneling probability themselves will be presented. For example, if $\log_{10}(T_{Q})=-6.5$, then $T_{Q}=10^{-6.5}=3.16\times 10^{-7}$.

The graph (a) of Figure 2 evaluates the quantum tunneling probability of extracellular protons (using the common logarithm) across the range of $E_{Gate}$ from 2.5 to 7 J and at three different setting values of gate length *L* (*L* = 1.5 m, *L* = 2 m, and *L* = 2.5 m). The evaluation is made by setting $V_{m}=0.087$ V and n = 1. See Table 1.

The graph (b) of Figure 2 evaluates the quantum tunneling probability of extracellular protons across the range of $E_{Gate}$ from 2.5 to 7 J and at three different setting values of membrane potential (*V_m_* = 0.087 V, *V_m_* = 0.077 V, and *V_m_* = 0.067 V). The evaluation is made by setting L=1.5 m and n = 1. See Table 2.

The graph (c) of Figure 2 evaluates the quantum tunneling probability of extracellular protons across the range of $E_{Gate}$ from 2.5 J and 7 J and at three different setting values of gate location (n = 1, n = 2, and n = 4). The evaluation is made by setting $V_{m}=0.087$ V and L=1.5 m. See Table 3.

The graph (d) of Figure 2 evaluates the quantum tunneling probability of intracellular protons across the range of $E_{Gate}$ from 1 to 7 J and at three different setting values of gate length *L* (*L* = 1.5 m, *L* = 2 m, and *L* = 2.5 m). See Table 4.

The graph (a) of Figure 3 evaluates the quantum tunneling probability of extracellular sodium ions across the range of $E_{Gate}$ from 2.5 to 7 J and at three different setting values of gate length *L* (*L* = 1.5 m, *L* = 2 m, and *L* = 2.5 m). The evaluation is made by setting $V_{m}=0.087$ V and n = 1. See Table 5.

The graph (b) of Figure 3 evaluates the quantum tunneling probability of extracellular sodium ions across the range of $E_{Gate}$ from 2.5 to 7 J and at three different setting values of membrane potential (V_m_ = 0.087 V, V_m_ = 0.077 V, and V_m_ = 0.067 V). The evaluation is made by setting L=1.5 m and n = 1. See Table 6.

The graph (c) of Figure 3 evaluates the quantum tunneling probability of extracellular sodium ions across the range of $E_{Gate}$ from 2.5 and 7 J and at three different setting values of gate location (n = 1, n = 2, and n = 4). The evaluation is made by setting $V_{m}=0.087$ V and L=1.5 m. See Table 7.

The graph (d) of Figure 3 evaluates the quantum tunneling probability of intracellular sodium ions across the range of $E_{Gate}$ from 1 to 7 J and at three different setting values of gate length *L* (*L* = 1.5 m, *L* = 2 m, and *L* = 2.5 m). See Table 8.

By comparing the tables of quantum tunneling of protons and sodium ions, it is clear that:

1. The tunneling probability of extracellular ions (protons and sodium ions) is higher than the tunneling probability of intracellular ions (protons and sodium ions) at the same setting values;
2. The tunneling probability of extracellular and intracellular protons is higher than the extracellular and intracellular sodium ions at the same setting values;
3. As the energy of the gate increases, the tunneling probability of ions (protons and sodium ions) decreases;
4. As the gate length increases, the tunneling probability of ions (protons and sodium ions) decreases;
5. As the absolute value of membrane potential (negative inside with regard to outside) increases, the tunneling probability of extracellular ions increases;
6. As the location of gate (n) increases, the value of membrane potential available for the kinetic energy of extracellular ions decreases and thus their tunneling probability decreases.

The previous discussion is also valid on quantum conductance of a single channel and the quantum membrane conductance because they are dependent on the tunneling probability, but the quantum membrane conductance is also dependent on the number of channels available for tunneling and these channels must either be in closed form (activation gate is closed) or inactivated (inactivation gate is closed) so that the principle of quantum tunneling can be applied, because when the channel is open, ions passage is predicted according to the classical mechanics.

Classically, when the voltage-gated sodium channel Nav1.5 is open, the single channel conductance for sodium ions is $17.3\times 10^{-12}$ S [8,27], and since protons have 252 times the permeability of sodium ions [28], it is expected that protons have single channel conductance $4.36\times 10^{-9}$ S. From the classical perspective, these channels either have these values of conductance for protons and sodium when they are open or they have zero values of conductance when they are closed (activation gate is closed) or inactivated (inactivation gate is closed). Interestingly, the quantum tunneling provides a continuous spectrum of quantum conductance values as presented in Figure 4 and Figure 5. This spectrum includes lower values than the classical values especially at higher values of gate energy $E_{Gate}$ and includes higher values than the classical values especially at lower values of gate energy $E_{Gate}$, which is the case during acidosis and its associated pathological processes. Moreover, the values of quantum conductance can be further changed by changing the other factors such as the length, membrane potential, and the gate’s location as presented in the graphs in Figure 4 and Figure 5. A numerical description of the quantum conductance of single channel for protons and sodium ions will be useful to make the results clear and comprehensible and to elucidate the differences between the classical conductance and the quantum conductance of a single channel. See the following tables.

The graph (a) of Figure 4 evaluates the quantum conductance of a single channel for extracellular protons across the range of $E_{Gate}$ from 2.5 to 7 J and at three different setting values of gate length *L* (*L* = 1.5 m, *L* = 2 m, and *L* = 2.5 m). The evaluation is made by setting $V_{m}=0.087$ V and n = 1. See Table 9.

The graph (b) of Figure 4 evaluates the quantum conductance of a single channel for extracellular protons across the range of $E_{Gate}$ from 2.5 to 7 J and at three different setting values of membrane potential (V_m_ = 0.087 V, V_m_ = 0.077 V, and V_m_ = 0.067 V). The evaluation is made by setting L=1.5 m and n = 1. See Table 10.

The graph (c) of Figure 4 evaluates the quantum conductance of a single channel for extracellular protons across the range of $E_{Gate}$ from 2.5 and 7 J and at three different setting values of gate location (n = 1, n = 2, and n = 4). The evaluation is made by setting $V_{m}=0.087$ V and L=1.5 m. See Table 11.

The graph (d) of Figure 4 evaluates the quantum conductance of a single channel for intracellular protons across the range of $E_{Gate}$ from 1 to 7 J and at three different setting values of gate length *L* (*L* = 1.5 m, *L* = 2 m, and *L* = 2.5 m). See Table 12.

By comparing the tables of quantum conductance of single channel for protons, it is clear that they do not have a single value of conductance as in the classical model, but a spectrum of continuous values spanning from the values calculated at $E_{Gate}=2.5$ J for extracellular protons and $E_{Gate}=1$ J for intracellular protons to $E_{Gate}=7$ J. Interestingly, both extracellular and intracellular protons, at 2.5 and 1 J, respectively, obtain quantum conductance values higher than the assigned classical conductance value, which is $4.36\times 10^{-9}$ S. This is true for all the values at 2.5 and 1 J. These higher quantum conductance values continue to decrease until reaching at $E_{Gate}=7$ J.

The graph (a) of Figure 5 evaluates the quantum conductance of a single channel for extracellular sodium ions across the range of $E_{Gate}$ from 2.5 to 7 J and at three different setting values of gate length *L* (*L* = 1.5 m, *L* = 2 m, and *L* = 2.5 m). The evaluation is made by setting $V_{m}=0.087$ V and n = 1. See Table 13.

The graph (b) of Figure 5 evaluates the quantum conductance of a single channel for extracellular sodium ions across the range of $E_{Gate}$ from 2.5 to 7 J and at three different setting values of membrane potential (V_m_ = 0.087 V, V_m_ = 0.077 V, and V_m_ = 0.067 V). The evaluation is made by setting L=1.5 m and n = 1. See Table 14.

The graph (c) of Figure 5 evaluates the quantum conductance of a single channel for extracellular sodium ions across the range of $E_{Gate}$ from 2.5 and 7 J and at three different setting values of gate location (n = 1, n = 2, and n = 4). The evaluation is made by setting $V_{m}=0.087$ V and L=1.5 m. See Table 15.

The graph (d) of Figure 5 evaluates the quantum conductance of a single channel for intracellular sodium ions across the range of $E_{Gate}$ from 1 to 7 J and at three different setting values of gate length *L* (*L* = 1.5 m, *L* = 2 m, and *L* = 2.5 m).See Table 16.

By comparing the tables of quantum conductance of single channel for sodium ions, it is clear that they do not have a single value of conductance as in the classical model, but a spectrum of continuous values spanning from the values calculated at $E_{Gate}=2.5$ J for extracellular sodium ions and $E_{Gate}=1$ J for intracellular sodium ions to $E_{Gate}=7$ J. Interestingly, both extracellular and intracellular sodium ions, at 2.5 and 1 J, respectively, can obtain quantum conductance values higher than the assigned classical conductance value, which is $17.3\times 10^{-12}$ S. However, this is not always true for all the values at 2.5 and 1 J because other factors such as gate length, membrane depolarization, and gate location modulate the values of quantum conductance of a single channel. These high quantum conductance values continue to decrease until reaching at $E_{Gate}=7$ J.

The previous six conclusions applied to the quantum tunneling probability of protons and sodium ions are also applied to the quantum conductance of a single channel of protons and sodium ions.

Furthermore, protons and sodium can achieve quantum membrane conductance higher than the conductance achieved classically by opening the same number of channels available for tunneling. This observation, as said before, is prominent at lower values of gate energy $E_{Gate}$. Again, protons achieve higher quantum membrane conductance than sodium ions due to the mass effect, and extracellular ions achieve higher quantum membrane conductance than the intracellular ions due to the discrepancy in their kinetic energies. This can be found in Figure 6 and Figure 7. A numerical description of the quantum membrane conductance of protons and sodium ions will be useful to make the results clear and comprehensible. See the following tables.

The graph (a) of Figure 6 evaluates the quantum membrane conductance of extracellular protons across the range of $E_{Gate}$ from 2.5 to 7 J and at three different setting values of gate length *L* (*L* = 1.5 m, *L* = 2 m, and *L* = 2.5 m). The evaluation is made by setting $V_{m}=0.087$ V, $D=10^{11}$ channels/cm^2^, and n = 1. See Table 17.

The graph (b) of Figure 6 evaluates the quantum membrane conductance of extracellular protons across the range of $E_{Gate}$ from 2.5 to 7 J and at three different setting values of membrane potential (V_m_ = 0.087 V, V_m_ = 0.077 V, and V_m_ = 0.067 V). The evaluation is made by setting L=1.5 m, $D=10^{11}$ channels/cm^2^, and n = 1. See Table 18.

The graph (c) of Figure 6 evaluates the quantum membrane conductance of extracellular protons across the range of $E_{Gate}$ from 2.5 and 7 J and at three different setting values of gate location (n = 1, n = 2, and n = 4). The evaluation is made by setting $V_{m}=0.087$ V, L=1.5 m, and $D=10^{11}$ channels/cm^2^. See Table 19.

The graph (d) of Figure 6 evaluates the quantum membrane conductance of extracellular protons across the range of $E_{Gate}$ from 2.5 to 7 J and at three different setting values of channels density ($D=10^{11}$ channels/cm^2^, $D=10^{10}$ channels/cm^2^, and $D=10^{9}$ channels/cm^2^). The evaluation is made by setting $V_{m}=0.087$ V, L=1.5 m, and n = 1. See Table 20.

The graph (e) of Figure 6 evaluates the quantum membrane conductance of intracellular protons across the range of $E_{Gate}$ from 1 to 7 J and at three different setting values of gate length *L* (*L* = 1.5 m, *L* = 2 m, and *L* = 2.5 m). The evaluation is made by setting $D=10^{11}$ channels/cm^2^. See Table 21.

The graph (f) of Figure 6 evaluates the quantum membrane conductance of intracellular protons across the range of $E_{Gate}$ from 1 to 7 J and at three different setting values of channels density ($D=10^{11}$ channels/cm^2^, $D=10^{10}$ channels/cm^2^, and $D=10^{9}$ channels/cm^2^). The evaluation is made by setting L=1.5 m. See Table 22.

The classical membrane conductance of protons can be calculated by multiplying the conductance of single channel $4.36\times 10^{-9}$ S by the density of channels D. Hence, the classical membrane conductance of protons is $4.36\times 10^{5}$ mS/cm^2^ at $D=10^{11}$ channels/cm^2^, $4.36\times 10^{4}$ mS/cm^2^ at $D=10^{10}$ channels/cm^2^, and $4.36\times 10^{3}$ mS/cm^2^ at $D=10^{9}$ channels/cm^2^. By comparing these values with the values in tables of protons, it is clear that both extracellular protons at 2.5 J and intracellular protons at 1 J obtain quantum membrane conductance higher than the classical membrane conductance. This is true for all the values at 2.5 and 1 J. These higher quantum membrane conductance values continue to decrease until reaching at $E_{Gate}=7$ J. The classical membrane conductance of protons mediated by voltage-gated sodium channels is valid only when the channels are open and the open voltage-gated sodium channels are not available at the resting state, during repolarization, or after repolarization because voltage-gated sodium channels in these stages are either closed or inactivated. On the other hand, the quantum membrane conductance of protons is valid when the channels are either closed or inactivated, which are prominently available during the resting state, repolarization phase, and after repolarization. Interestingly, it is clear from the tables that protons achieve quantum membrane conductance higher than the leaky classical membrane conductance of potassium and sodium ions (0.5 mS/cm^2^ and 0.005 mS/cm^2^, respectively) at the resting state. This predicts the ability of protons to depolarize the resting membrane potential via quantum tunneling since extracellular protons have higher quantum conductance than the intracellular protons and the intracellular to extracellular concentration ratio of protons, which is 10, is lower than the ratio of potassium ions, which is 140/4 = 35.

The graph (a) of Figure 7 evaluates the quantum membrane conductance of extracellular sodium ions across the range of $E_{Gate}$ from 2.5 to 7 J and at three different setting values of gate length *L* (*L* = 1.5 m, *L* = 2 m, and *L* = 2.5 m). The evaluation is made by setting $V_{m}=0.087$ V, $D=10^{11}$ channels/cm^2^, and n = 1. See Table 23.

The graph (b) of Figure 7 evaluates the quantum membrane conductance of extracellular sodium ions across the range of $E_{Gate}$ from 2.5 to 7 J and at three different setting values of membrane potential (V_m_ = 0.087 V, V_m_ = 0.077 V, and V_m_ = 0.067 V). The evaluation is made by setting L=1.5 m, $D=10^{11}$ channels/cm^2^, and n = 1. See Table 24.

The graph (c) of Figure 7 evaluates the quantum membrane conductance of extracellular sodium ions across the range of $E_{Gate}$ from 2.5 and 7 J and at three different setting values of gate location (n = 1, n = 2, and n = 4). The evaluation is made by setting $V_{m}=0.087$ V, L=1.5 m, and $D=10^{11}$ channels/cm^2^. See Table 25.

The graph (d) of Figure 7 evaluates the quantum membrane conductance of extracellular sodium ions across the range of $E_{Gate}$ from 2.5 to 7 J and at three different setting values of channels density ($D=10^{11}$ channels/cm^2^, $D=10^{10}$ channels/cm^2^, and $D=10^{9}$ channels/cm^2^). The evaluation is made by setting $V_{m}=0.087$ V, L=1.5 m, and n = 1. See Table 26.

The graph (e) of Figure 7 evaluates the quantum membrane conductance of intracellular sodium ions across the range of $E_{Gate}$ from 1 to 7 J and at three different setting values of gate length *L* (*L* = 1.5 m, *L* = 2 m, and *L* = 2.5 m). The evaluation is made by setting $D=10^{11}$ channels/cm^2^. See Table 27.

The graph (f) of Figure 7 evaluates the quantum membrane conductance of intracellular sodium ions across the range of $E_{Gate}$ from 1 to 7 J and at three different setting values of channels density ($D=10^{11}$ channels/cm^2^, $D=10^{10}$ channels/cm^2^, and $D=10^{9}$ channels/cm^2^). The evaluation is made by setting L=1.5 m. See Table 28.

The classical membrane conductance of sodium ions can be calculated by multiplying the conductance of single channel $17.3\times 10^{-12}$ S by the density of channels D. Hence, the classical membrane conductance of sodium ions is $17.3\times 10^{2}$ mS/cm^2^ at $D=10^{11}$ channels/cm^2^, 173 mS/cm^2^ at $D=10^{10}$ channels/cm^2^, and 17.3 mS/cm^2^ at $D=10^{9}$ channels/cm^2^. By comparing these values with the values in tables of sodium ions, it is clear that both extracellular sodium ions at 2.5 J and intracellular sodium ions at 1 J can obtain quantum membrane conductance higher than the classical membrane conductance. This is not true for all the values at 2.5 and 1 J because other factors such as gate length, membrane potential, and the gate location modulate the values of quantum membrane conductance. These high quantum membrane conductance values continue to decrease until reaching at $E_{Gate}=7$ J. The classical membrane conductance of sodium ions mediated by voltage-gated sodium channels is valid only when the channels are open and the open voltage-gated sodium channels are not available at the resting state, during repolarization, or after repolarization because voltage-gated sodium channels in these stages are either closed or inactivated. On the other hand, the quantum membrane conductance of sodium ions is valid when the channels are either closed or inactivated, which are prominently available during the resting state, repolarization phase, and after repolarization. Interestingly, it is clear from the tables that sodium ions achieve quantum membrane conductance higher than the leaky classical membrane conductance of potassium and sodium ions (0.5 mS/cm^2^ and 0.005 mS/cm^2^, respectively) at the resting state. This predicts the ability of sodium ions to depolarize the resting membrane potential via quantum tunneling, since extracellular sodium ions have higher quantum conductance than the intracellular sodium ions and the extracellular concentration of sodium ions is higher than their intracellular concentration.

The previous six conclusions applied to quantum tunneling probability and quantum conductance of single channel are also applied to the quantum membrane conductance of protons and sodium ions.

In the previous discussion, we focused on the tunneling probability and quantum conductance because conductance is a crucial factor that affects membrane potential. According to the GHK equation, membrane potential is influenced by two main factors: (1) the ion’s concentration and (2) the ion’s conductance or permeability. When the quantum conductance is inserted into the classical version of the GHK equation, we obtain a quantum version. This equation calculates the membrane potential at resting state, at which the channels are in a closed state or an inactivated state. The percentages of these states depend on the membrane potential. As the membrane potential depolarizes, the number of inactivated channels increases and the number of closed channels (activation gate is closed) decreases. However, both states can be used to apply the model of quantum tunneling of protons. This equation can give reflection about the degree of depolarization according to certain values of concentration and conductance. Hence, it is used in this study to assess the effect of quantum conductance of protons on the membrane potential and the degree of depolarization induced by the quantum tunneling of protons. It is obvious from Figure 8 that protons are able to depolarize the membrane potential and this depolarization becomes more apparent as the energy of gate $E_{Gate}$ decreases. Interestingly, the very low concentration of protons (when it is compared with sodium ions) is compensated by the high quantum membrane conductance of protons. This is the reason behind the direct effect of depolarization mediated by protons themselves despite the minute concentration. The quantum model suggests that protons can depolarize the membrane potential directly and not only through indirect effects on the channels or indirect events such as hyperkalemia. This distinguishes the quantum mechanism from other mechanisms. Besides, other factors such as the gate’s length and channel’s density modulate the degree of depolarization, and this is expected because it was explained in the case of quantum conductance and quantum tunneling. In addition to that, low extracellular pH during acidosis is a contributing factor to the depolarization because low extracellular pH means higher concentration of protons and consequently larger flow to inside the cell via quantum tunneling. Notably in Figure 9, sodium ions can depolarize the membrane potential by their quantum tunneling and quantum conductance at lower values of gate’s energy. Even though sodium ions have less tunneling probability and less quantum conductance compared to protons, the concentration of extracellular sodium ions (142 mEq/L) is much higher than the extracellular protons ($3.98\times 10^{-5}$ mEq/L, at pH = 7.4), so lower tunneling probability and lower quantum conductance are required for sodium ions to show a depolarization effect. If Figure 8 and Figure 9 are compared, it is obvious that protons can depolarize the resting membrane potential at higher values of $E_{Gate}$ in comparison with sodium ions. This indicates that protons contribute to the pathogenesis of arrhythmias at early stages of pathologies and they are exacerbated by the quantum tunneling of sodium ions at later stages as the diseases progress and the clinical status deteriorates. Thus, the lower values of $E_{Gate}$ for activation and inactivation gate during acidosis enhance the quantum tunneling of both protons and sodium ions through these closed gates. This enhancement results in significant quantum conductance that can depolarize the membrane potential. The other factors will further modulate the degree of depolarization. In Figure 8 and Figure 9, it is clear that there is a plateau (high plateau) at membrane potential of 0.087 V, which is the original membrane potential. This indicates that the decrease in the energy of the gate across this plateau is not enough to affect the original membrane potential via quantum tunneling. Additionally, the plateau in the graphs of protons is shorter than the plateau in the graphs of sodium ions. This means that protons are able to depolarize the resting membrane potential at higher values of energy of gate $E_{Gate}$ as we said before. In other words, protons are more sensitive to the drop in the energy of gate $E_{Gate}$ if it they are compared with sodium ions. On the other hand, protons have another plateau (low plateau) at the end of the graphs of Figure 8, which is above the 0 V of membrane potential, while sodium ions do not have such a plateau and continue to depolarize the membrane potential until reaching 0 V as in Figure 9. This may be attributed to the higher extracellular sodium concentration than the intracellular concentration, which guarantees sodium ions to continue in membrane depolarization until reaching 0 V, while the intracellular concentration of protons is higher than the extracellular concentration, which prevents protons to reach 0 V.

To make the differences between protons and sodium ions clear in terms of membrane depolarization, a numerical comparison based on Figure 8 and Figure 9 will be useful.

In the following tables, we are going to use two quantities to make the results clear and comprehensible. These two quantities are the point of curving $E_{cur}$ and the average rate of depolarization in membrane potential with respect to change in the energy of gate $E_{Gate}$ (V/J). The point of curving is defined as the value of $E_{Gate}$ at which the membrane potential becomes 0.086 V (dropping from 0.087 to 0.086). We describe this value as point of curving because the relationship between the energy of the gate and membrane potential begins as a plateau at the original membrane potential 0.087 V, and the beginning of the drop in membrane potential represents the beginning of the curving from the plateau. See Figure 8 and Figure 9. We choose the value 0.086 V as the membrane potential for the point of curving to be the reference value that can be applied on all the graphs. The point of curving can be found by substituting the membrane potential as 0.086 V and substituting all the other setting values of each graph in Equation (21) for protons and Equation (22) for sodium ions, then solving the equation for $E_{Gate}$. The solution value of $E_{Gate}$ is called point of curving $E_{cur}$. The point of curving will be used to differentiate between the effect of quantum tunneling of protons and sodium ions on the resting membrane potential because the point of curving gives a reflection about the sensitivity of ions to depolarize the membrane potential with respect to the drop in the energy of gate $E_{Gate}$. The higher the value of point of curving, the more sensitive the ion to depolarize the membrane potential with respect to the drop in the energy of the gate. On the other hand, the average rate of depolarization of membrane potential relative to change in the energy of the gate gives a reflection about how fast the ions are able to depolarize the membrane potential with respect to the change in the energy of the gate.

The following tables will investigate the properties of protons to depolarize the membrane potential. Moreover, the average rate of change will be calculated from the point of curving to the end of the graph, which is at $E_{Gate}=1$. See Figure 8.

The average rate of depolarization for protons can be calculated by the following equation:

(26) $$R(H)=\frac{\Delta V_{m}}{\Delta E_{Gate}}=\frac{V_{m(1)}-V_{m(cur)}}{1-E_{cur}}=\frac{V_{m(1)}-0.086}{1-E_{cur}},$$

where, R(H) is the average rate of depolarization for protons, $V_{m(1)}$ is the membrane potential at $E_{Gate}=1$, $V_{m(cur)}$ is the membrane potential at the point of curving $E_{cur}$, which is 0.086 V.

The graph (a) of Figure 8 evaluates the effect of the energy of gate $E_{Gate}$ on the resting membrane potential under the influence of quantum tunneling of protons at three different values of $pH_{E}$ ($pH_{E}=7.4$, $pH_{E}=7$, $pH_{E}=6.5$) and by setting L=1.5 m, $D=10^{11}$ channels/cm^2^, and n = 1. See Table 29.

From Table 29, as the $pH_{E}$ decreases, the point of curving $E_{cur}(H)$ increases. This means protons are more sensitive to the drop in the energy of the gate at lower values of $pH_{E}$ and the depolarization occurs at higher values of energy of the gate. This is manifested as shorter high plateau at lower values of $pH_{E}$, see Figure 8. The membrane potential at $E_{Gate}=1$ J is almost the same for all $pH_{E}$ values. Moreover, as the value of $pH_{E}$ decreases, the average rate of depolarization decreases. In other words, as the protons become more sensitive to the drop in the energy of the gate (which is associated with lower values of $pH_{E}$), the average rate of depolarization becomes slower.

The graph (b) of Figure 8 evaluates the effect of the energy of the gate $E_{Gate}$ on the resting membrane potential under the influence of quantum tunneling of protons at three different values of gate length (L=1.5 m, L=2 m, and L=2.5 m) and by setting $pH_{E}=7.4$, $D=10^{11}$ channels/cm^2^, and n = 1. See Table 30.

From Table 30, as the gate length *L* increases, the point of curving $E_{cur}(H)$ decreases. This means protons are more sensitive to the drop in the energy of gate at lower values of gate length *L* and the depolarization occurs at higher values of energy of the gate. This is manifested as a shorter high plateau at lower values of gate length *L*, see Figure 8. Additionally, as the gate length *L* increases, the membrane potential becomes more depolarized at $E_{Gate}=1$ J. Moreover, as the value of gate length *L* increases, the average rate of depolarization increases. In other words, as the protons become more sensitive to the drop in the energy of the gate (which is associated with lower values of gate length *L*), the average rate of depolarization becomes slower.

The graph (c) of Figure 8 evaluates the effect of the energy of gate $E_{Gate}$ on the resting membrane potential under the influence of quantum tunneling of protons at three different values of channels density ($D=10^{11}$ channels/cm^2^, $D=10^{10}$ channels/cm^2^, and $D=10^{9}$ channels/cm^2^) and by setting $pH_{E}=7.4$, L=1.5 m, and n = 1. See Table 31.

From Table 31, as the density of channels decreases, the point of curving $E_{cur}(H)$ decreases. This means protons are more sensitive to the drop in the energy of the gate at higher values of channels density and the depolarization occurs at higher values of energy of the gate. This is manifested as a shorter high plateau at higher values of channels density; see Figure 8. Additionally, the membrane potential at $E_{Gate}=1$ J is almost the same for all values of channels density. Moreover, as the value of channels density decreases, the average rate of depolarization increases. In other words, as the protons become more sensitive to the drop in the energy of the gate (which is associated with higher values of channels density), the average rate of depolarization becomes slower.

The graph (d) of Figure 8 evaluates the effect of the energy of gate $E_{Gate}$ on the resting membrane potential under the influence of quantum tunneling of protons at three different values of gate location (n = 1, n = 2, and n = 4) and by setting $pH_{E}=7.4$, L=1.5 m, and $D=10^{11}$ channels/cm^2^. See Table 32.

From Table 32, as the location of the gate (n) increases, the point of curving $E_{cur}(H)$ decreases. This means protons are more sensitive to the drop in the energy of the gate at lower values of gate location (n) and the depolarization occurs at higher values of energy of the gate. This is manifested as a shorter high plateau at lower values of gate location (n), see Figure 8. Additionally, as the gate location (n) increases, the membrane potential becomes less depolarized at $E_{Gate}=1$ J. Moreover, as the value of gate location (n) increases, the average rate of depolarization increases. In other words, as the protons become more sensitive to the drop in the energy of the gate (which is associated with lower values of gate location (n)), the average rate of depolarization becomes slower.

In the following tables, we are going to investigate the depolarization induced by the quantum tunneling of sodium ions. However, it seems obvious from Figure 9 that sodium ions are able to depolarize the membrane potential to 0 V. Therefore, in case of sodium ions, we will focus on the values of $E_{Gate}$ at which the membrane potential is 0 V. The reason behind this is we want to investigate from the point of curving to the end of graph, which is at the 0 V. On the other hand, the end of the graph, in case of protons, is at $E_{Gate}=1$ J as presented before in the tables of protons.

The average rate of depolarization for sodium ions can be calculated by the following equation:

(27) $$R(Na)=\frac{\Delta V_{m}}{\Delta E_{Gate}}=\frac{0-V_{m(cur)}}{E_{Gate(0)}-E_{cur}}=\frac{-0.086}{E_{Gate(0)}-E_{cur}},$$

where R(Na) is the average rate of depolarization for sodium ions, $V_{m(cur)}$ is the membrane potential at the point of curving $E_{cur}$, and $E_{Gate(0)}$ is the energy of gate at $V_{m}=0$ V.

The graph (a) of Figure 9 evaluates the effect of the energy of gate $E_{Gate}$ on the resting membrane potential under the influence of quantum tunneling of sodium ions at three different values of gate length (L=1.5 m, L=2 m, and L=2.5 m) and by setting $D=10^{11}$ channels/cm^2^, and n = 1. See Table 33.

From Table 33, as the gate length *L* increases, the point of curving $E_{cur}(Na)$ decreases. This means sodium ions are more sensitive to the drop in the energy of the gate at lower values of gate length *L* and the depolarization occurs at higher values of energy of the gate. This is manifested as a shorter plateau at lower values of gate length *L*, see Figure 9. Additionally, as the gate length *L* increases, sodium ions reach the 0 V at lower values of energy of the gate. Moreover, as the value of gate length *L* increases, the average rate of depolarization increases. In other words, as sodium ions become more sensitive to the drop in the energy of the gate (which is associated with lower values of gate length *L*), the average rate of depolarization becomes slower.

The graph (b) of Figure 9 evaluates the effect of the energy of gate $E_{Gate}$ on the resting membrane potential under the influence of quantum tunneling of sodium ions at three different values of channels density ($D=10^{11}$ channels/cm^2^, $D=10^{10}$ channels/cm^2^, and $D=10^{9}$ channels/cm^2^) and by setting L=1.5 m, and n = 1. See Table 34.

From Table 34, as the density of channels decreases, the point of curving $E_{cur}(Na)$ decreases. This means sodium ions are more sensitive to the drop in the energy of the gate at higher values of channels density and the depolarization occurs at higher values of energy of the gate. This is manifested as shorter plateau at higher values of channels density; see Figure 9. Additionally, as the channels density decreases, sodium ions reach the 0 V at lower values of energy of gate. Moreover, as the value of channels density decreases, the average rate of depolarization increases. In other words, as sodium ions become more sensitive to the drop in the energy of the gate (which is associated with higher values of channels density), the average rate of depolarization becomes slower.

The graph (c) of Figure 9 evaluates the effect of the energy of gate $E_{Gate}$ on the resting membrane potential under the influence of quantum tunneling of sodium ions at three different values of gate location (n = 1, n = 2, and n = 4) and by setting L=1.5 m, and $D=10^{11}$ channels/cm^2^. See Table 35.

From Table 35, as the location of the gate (n) increases, the point of curving $E_{cur}(Na)$ decreases. This means sodium ions are more sensitive to the drop in the energy of the gate at lower values of gate location (n) and the depolarization occurs at higher values of energy of the gate. This is manifested as a shorter plateau at lower values of gate location (n), see Figure 9. Additionally, sodium ions reach the 0 V at almost the same value of energy of the gate. Moreover, as the value of gate location (n) increases, the average rate of depolarization increases. In other words, as sodium ions become more sensitive to the drop in the energy of the gate (which is associated with lower values of gate location (n)), the average rate of depolarization becomes slower.

By comparing the tables of depolarization for both protons and sodium ions, it is clear that protons are more sensitive than sodium ions to the drop in the energy of the gate. This is evident by the higher values of point of curving for protons $E_{cur}(H)$ if they are compared with the values of point of curving for sodium ions $E_{cur}(Na)$. However, sodium ions have a faster average rate of depolarization compared with protons.

Figure 10 shows the depolarization induced by the flow of protons through open sodium channels. The depolarization effect occurs when the open channels are available. However, this does not happen at the resting state or during the repolarization phase of action potential because sodium channels are either closed or inactivated but not open, except during the spike phase of action potential where sodium channels are mainly open for a short period of time. Furthermore, when protons flow through open channels during the spike, this will not affect the action potential because it is expected, according to the graph in Figure 10, that the flow of protons will depolarize the membrane potential which is already depolarizing during this phase of action potential. Consequently, the adverse effects of depolarization, which results into arrhythmias, occurs during resting state, repolarization phase, and following repolarization [4]. The channels during these phases are either inactivated or closed, but not open. This means that open channels are not available for the classical flow of protons to depolarize the membrane potential and to develop arrhythmias, but the closed and inactivated channels are available for quantum tunneling to happen and to develop arrhythmias mediated by depolarization. This is a vital distinction between the classical and quantum effects of protons. If Figure 8 and Figure 10 are compared, another distinction can be made. This distinction is that quantum effect of protons can depolarize the membrane potential much more than the classical effect of protons. In addition to that, the conductance is the same for extracellular and intracellular ions in the classical model, but the quantum conductance of extracellular protons is higher than that of intracellular protons. Hence, the quantum behavior of protons has a higher tendency to develop an inward current, unlike the classical behavior, which has less tendency to produce an inward current and may produce an outward current of protons, especially at high intracellular concentration or when the membrane potential is depolarized in a way that its absolute value is less than the absolute value of Nernst potential of protons.

The graph of Figure 10 evaluates the effect of $pH_{E}$ on the resting membrane potential under the influence of classical transport of protons though open sodium channels at three different values of channels density ($D=10^{11}$ channels/cm^2^, $D=10^{10}$ channels/cm^2^, and $D=10^{9}$ channels/cm^2^). The evaluation is made by setting the permeability ratio $P_{H}/P_{Na}=252$ and the classical single channel conductance of sodium channel $C_{Na}=17.3\times 10^{-12}$ S. See Table 36.

From Table 36, as the number of open channels increases, the membrane potential becomes more depolarized according to the classical electrochemical gradient of protons. Additionally, the graphs in Figure 10 tend to plateau at a membrane potential of 0.0615 V, which is the Nernst potential of protons at intracellular to extracellular concentration ratio of 10 that is the assumed ratio in this study. Therefore, when the open channels are available, protons cannot depolarize the membrane potential beyond their Nernst potential because both extracellular and intracellular have the same membrane conductance. On the other hand, the quantum transport of protons allows them to depolarize the membrane potential beyond the Nernst potential, as evident from the tables and figures. This is because extracellular protons have a higher quantum membrane conductance if they are compared with intracellular protons and this allows protons to have a higher ability to generate inward current to produce larger depolarization beyond the Nernst potential.

Even though our study focuses on the resting state with its corresponding conductance values of sodium and potassium ions, the depolarization induced by quantum tunneling is also valid during the repolarization phase and after repolarization because protons and even sodium ions showed a strong tendency to depolarize the membrane potential due to the significant quantum conductance as presented in the mathematical graphs in the results section. Therefore, we use the GHK equation to give an insight about the ability of protons to depolarize the membrane potential when the closed or inactivated channels are available.

In this context, we propose that gating pore currents of protons (omega currents) might be quantum currents through closed channels and the effects of mutations of S4 segments and acidosis are similar and result in decreasing the energy barrier of closed gates, enhancing the quantum tunneling of protons and other ions. Additionally, the ability of protons to pass through closed voltage-gated proton channels [29] supports the hypothesis that links gating pore currents and quantum currents. Interestingly, gating pore currents are inward currents [29,30], and this is more consistent with the prediction of the quantum model than the classical model. This proposal needs further studies and investigations, however, it is mentioned here because the quantum model predicts proton leakiness through an unusual pathway of permeation (through closed gates), which is characteristic of omega currents [30].

**Figure 14:**
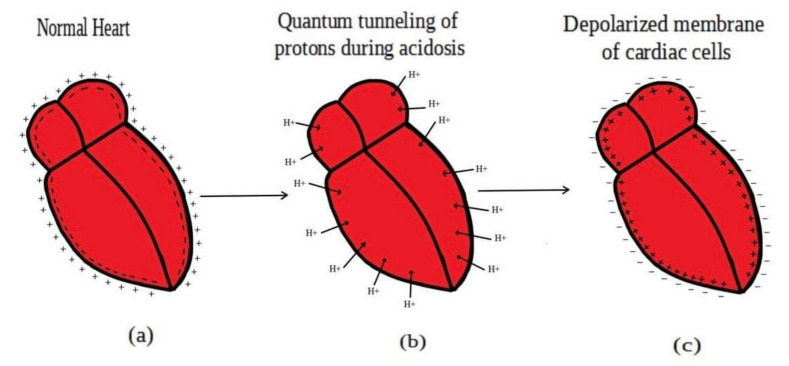
(**a**): represents normal heart with normal polarization (negative inside with regard to outside); (**b**): represents inward quantum tunneling of protons, which is indicated by inward arrows. This inward tunneling is responsible for membrane depolarization during acidosis according to the quantum model; (**c**): represents the state of membrane depolarization (positive inside with regard to outside), which is the outcome of protons tunneling that increases the tendency of arrhythmias and cardiac arrest.

To sum up, acidosis augments the quantum tunneling of protons resulting in a positive inward current that depolarizes the membrane potential. This state of membrane depolarization increases the tendency of cardiac arrhythmias or even cardiac arrest depending on the degree of depolarization. See Figure 14.

## Figures and Tables

**Table 1:** Represents the values of quantum tunneling probability of extracellular protons that take the range between the two values calculated at $E_{Gate}=2.5$ J and $E_{Gate}=7$ J. The evaluation is made at *L* = 1.5 m, *L* = 2 m, and *L* = 2.5 m and by setting $V_{m}=0.087$ V and n = 1.

The Gate Length *L* (m)	$T_{Q}{\left(H\right)}_{E}$ $\;\mathrm{at}\;E_{Gate}=2.5\;J$	$T_{Q}{\left(H\right)}_{E}$ $\;\mathrm{at}\;E_{Gate}=7\;J$
1.5	0.24	$2.6\times 10^{-8}$
2	0.15	$9.3\times 10^{-11}$
2.5	0.094	$2.9\times 10^{-13}$

**Table 2:** Represents the values of quantum tunneling probability of extracellular protons that take the range between the two values calculated at $E_{Gate}=2.5$ J and $E_{Gate}=7$ J. The evaluation is made at $V_{m}=0.087$ V, $V_{m}=0.077$ V and $V_{m}=0.067$ V and by setting *L* = 1.5 m and n = 1.

The Membrane Potential *V_m_* (V)	$T_{Q}{\left(H\right)}_{E}$ $\mathrm{at}\;E_{Gate}=2.5\;J$	$T_{Q}{\left(H\right)}_{E}$ $\mathrm{at}\;E_{Gate}=7\;J$
0.087	0.24	$2.6\times 10^{-8}$
0.077	0.11	$1.1\times 10^{-8}$
0.067	0.045	$4.7\times 10^{-9}$

**Table 3:** Represents the values of quantum tunneling probability of extracellular protons that take the range between the two values calculated at $E_{Gate}=2.5$ J and $E_{Gate}=7$ J. The evaluation is made at n = 1, n = 2, and n = 4 and by setting $V_{m}=0.087$ V and L=1.5 m.

The Location of Gate n	$T_{Q}{\left(H\right)}_{E}$ $\;\mathrm{at}\;E_{Gate}=2.5\;J$	$T_{Q}{\left(H\right)}_{E}$ $\;\mathrm{at}\;E_{Gate}=7\;J$
1	0.24	$2.6\times 10^{-8}$
2	$4\times 10^{-3}$	$6.9\times 10^{-10}$
4	$2.8\times 10^{-4}$	$8.3\times 10^{-11}$

**Table 4:** Represents the values of quantum tunneling probability of intracellular protons that take the range between the two values calculated at $E_{Gate}=1$ J and $E_{Gate}=7$ J. The evaluation is made at *L* = 1.5 m, *L* = 2 m, and *L* = 2.5 m.

The Gate Length *L* (m)	$T_{Q}{\left(H\right)}_{I}$ $\;\mathrm{at}\;E_{Gate}=1\;J$	$T_{Q}{\left(H\right)}_{I}$ $\;\mathrm{at}\;E_{Gate}=7\;J$
1.5	0.092	$1.1\times 10^{-11}$
2	0.042	$2.4\times 10^{-15}$
2.5	0.019	$5.1\times 10^{-19}$

**Table 5:** Represents the values of quantum tunneling probability of extracellular sodium ions that take the range between the two values calculated at $E_{Gate}=2.5$ J and $E_{Gate}=7$ J. The evaluation is made at *L* = 1.5 m, *L* = 2 m, and *L* = 2.5 m and by setting $V_{m}=0.087$ V and n = 1.

The Gate Length *L* (m)	$T_{Q}{\left(Na\right)}_{E}$ $\;\mathrm{at}\;E_{Gate}=2.5\;J$	$T_{Q}{\left(Na\right)}_{E}$ $\;\mathrm{at}\;E_{Gate}=7\;J$
1.5	$1.2\times 10^{-3}$	$8.1\times 10^{-37}$
2	$1.2\times 10^{-4}$	$7.6\times 10^{-49}$
2.5	$1.3\times 10^{-5}$	$7.1\times 10^{-61}$

**Table 6:** Represents the values of quantum tunneling probability of extracellular sodium ions that take the range between the two values calculated at $E_{Gate}=2.5$ J and $E_{Gate}=7$ J. The evaluation is made at $V_{m}=0.087$ V, $V_{m}=0.077$ V and $V_{m}=0.067$ V and by setting *L* = 1.5 m and n = 1.

The Membrane Potential *V_m_* (V)	$T_{Q}{\left(Na\right)}_{E}$ $\;\mathrm{at}\;E_{Gate}=2.5\;J$	$T_{Q}{\left(Na\right)}_{E}$ $\;\mathrm{at}\;E_{Gate}=7\;J$
0.087	$1.2\times 10^{-3}$	$8.1\times 10^{-37}$
0.077	$2.3\times 10^{-5}$	$1.4\times 10^{-38}$
0.067	$3.9\times 10^{-7}$	$2.3\times 10^{-40}$

**Table 7:** Represents the values of quantum tunneling probability of extracellular sodium ions that take the range between the two values calculated at $E_{Gate}=2.5$ J and $E_{Gate}=7$ J. The evaluation is made at n = 1, n = 2, and n = 4 and by setting $V_{m}=0.087$ V and L=1.5 m.

The Location of Gate n	$T_{Q}{\left(Na\right)}_{E}$ $\;\mathrm{at}\;E_{Gate}=2.5\;J$	$T_{Q}{\left(Na\right)}_{E}$ $\;\mathrm{at}\;E_{Gate}=7\;J$
1	$1.2\times 10^{-3}$	$8.1\times 10^{-37}$
2	$4\times 10^{-12}$	$1.4\times 10^{-44}$
4	$1.2\times 10^{-17}$	$1\times 10^{-48}$

**Table 8:** Represents the values of quantum tunneling probability of intracellular sodium ions that take the range between the two values calculated at $E_{Gate}=1$ J and $E_{Gate}=7$ J. The evaluation is made at *L* = 1.5 m, *L* = 2 m, and *L* = 2.5 m.

The Gate Length *L* (m)	$T_{Q}{\left(Na\right)}_{I}$ $\;\mathrm{at}\;E_{Gate}=1\;J$	$T_{Q}{\left(Na\right)}_{I}$ $\;\mathrm{at}\;E_{Gate}=7\;J$
1.5	$1.2\times 10^{-5}$	$5.7\times 10^{-53}$
2	$2.7\times 10^{-7}$	$2.2\times 10^{-70}$
2.5	$6.2\times 10^{-9}$	$8.5\times 10^{-88}$

**Table 9:** Represents the values of quantum conductance of single channel for extracellular protons that take the range between the two values calculated at $E_{Gate}=2.5$ J and $E_{Gate}=7$ J. The evaluation is made at *L* = 1.5 m, *L* = 2 m, and *L* = 2.5 m and by setting $V_{m}=0.087$ V and n = 1.

The Gate Length *L* (m)	$C_{Q}{\left(H\right)}_{E}$ $\;\mathrm{at}\;E_{Gate}=2.5\;J$	$C_{Q}{\left(H\right)}_{E}$ $\;\mathrm{at}\;E_{Gate}=7\;J$
1.5	$9.4\times 10^{-6}$ S	$1\times 10^{-12}$ S
2	$5.8\times 10^{-6}$ S	$3\times 10^{-15}$ S
2.5	$3.6\times 10^{-6}$ S	$9\times 10^{-18}$ S

**Table 10:** Represents the values of quantum conductance of single channel for extracellular protons that take the range between the two values calculated at $E_{Gate}=2.5$ J and $E_{Gate}=7$ J. The evaluation is made at $V_{m}=0.087$ V, $V_{m}=0.077$ V and $V_{m}=0.067$ V and by setting *L* = 1.5 m and n = 1.

The Membrane Potential *V_m_* (V)	$C_{Q}{\left(H\right)}_{E}$ $\;\mathrm{at}\;E_{Gate}=2.5\;J$	$C_{Q}{\left(H\right)}_{E}$ $\;\mathrm{at}\;E_{Gate}=7\;J$
0.087	$9.4\times 10^{-6}$ S	$1\times 10^{-12}$ S
0.077	$4.3\times 10^{-6}$ S	$4.3\times 10^{-13}$ S
0.067	$1.8\times 10^{-6}$ S	$1.8\times 10^{-13}$ S

**Table 11:** Represents the values of quantum conductance of single channel for extracellular protons that take the range between the two values calculated at $E_{Gate}=2.5$ J and $E_{Gate}=7$ J. The evaluation is made at n = 1, n = 2, and n = 4 and by setting $V_{m}=0.087$ V and L=1.5 m.

The Location of Gate n	$C_{Q}{\left(H\right)}_{E}$ $\;\mathrm{at}\;E_{Gate}=2.5\;J$	$C_{Q}{\left(H\right)}_{E}$ $\;\mathrm{at}\;E_{Gate}=7\;J$
1	$9.4\times 10^{-6}$ S	$1\times 10^{-12}$ S
2	$1.6\times 10^{-7}$ S	$2.4\times 10^{-14}$ S
4	$1.1\times 10^{-8}$ S	$3.2\times 10^{-15}$ S

**Table 12:** Represents the values of quantum conductance of a single channel for intracellular protons that take the range between the two values calculated at $E_{Gate}=1$ J and $E_{Gate}=7$ J. The evaluation is made at *L* = 1.5 m, *L* = 2 m, and *L* = 2.5 m.

The Gate Length *L* (m)	$C_{Q}{\left(H\right)}_{I}$ $\;\mathrm{at}\;E_{Gate}=1\;J$	$C_{Q}{\left(H\right)}_{I}$ $\;\mathrm{at}\;E_{Gate}=7\;J$
1.5	$3.6\times 10^{-6}$ S	$4.1\times 10^{-16}$ S
2	$1.6\times 10^{-6}$ S	$9.1\times 10^{-20}$ S
2.5	$7.3\times 10^{-7}$ S	$2\times 10^{-23}$ S

**Table 13:** Represents the values of quantum conductance of single channel for extracellular sodium ions that take the range between the two values calculated at $E_{Gate}=2.5$ J and $E_{Gate}=7$ J. The evaluation is made at *L* = 1.5 m, *L* = 2 m, and *L* = 2.5 m and by setting $V_{m}=0.087$ V and n = 1.

The Gate Length *L* (m)	$C_{Q}{\left(Na\right)}_{E}$ $\;\mathrm{at}\;E_{Gate}=2.5\;J$	$C_{Q}{\left(Na\right)}_{E}$ $\;\mathrm{at}\;E_{Gate}=7\;J$
1.5	$4.5\times 10^{-8}$ S	$3.2\times 10^{-41}$ S
2	$4.7\times 10^{-9}$ S	$2.9\times 10^{-53}$ S
2.5	$4.9\times 10^{-10}$ S	$2.8\times 10^{-65}$ S

**Table 14:** Represents the values of quantum conductance of single channel for extracellular sodium ions that take the range between the two values calculated at $E_{Gate}=2.5$ J and $E_{Gate}=7$ J. The evaluation is made at $V_{m}=0.087$ V, $V_{m}=0.077$ V and $V_{m}=0.067$ V and by setting *L* = 1.5 m and n = 1.

The Membrane Potential *V_m_* (V)	$C_{Q}{\left(Na\right)}_{E}$ $\;\mathrm{at}\;E_{Gate}=2.5\;J$	$C_{Q}{\left(Na\right)}_{E}$ $\;\mathrm{at}\;E_{Gate}=7\;J$
0.087	$4.5\times 10^{-8}$ S	$3.2\times 10^{-41}$ S
0.077	$1.1\times 10^{-9}$ S	$5.5\times 10^{-43}$ S
0.067	$1.5\times 10^{-11}$ S	$9.1\times 10^{-45}$ S

**Table 15:** Represents the values of quantum conductance of single channel for extracellular sodium ions that take the range between the two values calculated at $E_{Gate}=2.5$ J and $E_{Gate}=7$ J. The evaluation is made at n = 1, n = 2, and n = 4 and by setting $V_{m}=0.087$ V and L=1.5 m.

The Location of Gate n	$C_{Q}{\left(Na\right)}_{E}$ $\;\mathrm{at}\;E_{Gate}=2.5\;J$	$C_{Q}{\left(Na\right)}_{E}$ $\;\mathrm{at}\;E_{Gate}=7\;J$
1	$4.5\times 10^{-8}$ S	$3.2\times 10^{-41}$ S
2	$1.6\times 10^{-16}$ S	$5.4\times 10^{-49}$ S
4	$4.6\times 10^{-22}$ S	$4\times 10^{-53}$ S

**Table 16:** Represents the values of quantum conductance of single channel for intracellular sodium ions that take the range between the two values calculated at $E_{Gate}=1$ J and $E_{Gate}=7$ J. The evaluation is made at *L* = 1.5 m, *L* = 2 m, and *L* = 2.5 m.

The Gate Length *L* (m)	$C_{Q}{\left(Na\right)}_{I}$ $\;\mathrm{at}\;E_{Gate}=1\;J$	$C_{Q}{\left(Na\right)}_{I}$ $\;\mathrm{at}\;E_{Gate}=7\;J$
1.5	$4.6\times 10^{-10}$ S	$2.2\times 10^{-57}$ S
2	$1.1\times 10^{-11}$ S	$8.5\times 10^{-75}$ S
2.5	$2.4\times 10^{-13}$ S	$3.3\times 10^{-92}$ S

**Table 17:** Represents the values of quantum membrane conductance of extracellular protons that take the range between the two values calculated at $E_{Gate}=2.5$ J and $E_{Gate}=7$ J. The evaluation is made at *L* = 1.5 m, *L* = 2 m, and *L* = 2.5 m and by setting $V_{m}=0.087$ V, $D=10^{11}$ channels/cm^2^, and n = 1.

The Gate Length *L* (m)	$MC_{Q}{\left(H\right)}_{E}$ $\;\mathrm{at}\;E_{Gate}=2.5\;J$	$MC_{Q}{\left(H\right)}_{E}$ $\;\mathrm{at}\;E_{Gate}=7\;J$
1.5	$9.4\times 10^{8}$ mS/cm^2^	101.5 ms/cm^2^
2	$5.8\times 10^{8}$ mS/cm^2^	0.3 mS/cm^2^
2.5	$3.6\times 10^{8}$ mS/cm^2^	$9\times 10^{-4}$ mS/cm^2^

**Table 18:** Represents the values of quantum membrane conductance of extracellular protons that take the range between the two values calculated at $E_{Gate}=2.5$ J and $E_{Gate}=7$ J. The evaluation is made at $V_{m}=0.087$ V, $V_{m}=0.077$ V and $V_{m}=0.067$ V and by setting *L* = 1.5 m, $D=10^{11}$ channels/cm^2^, and n = 1.

The Membrane Potential *V_m_* (V)	$MC_{Q}{\left(H\right)}_{E}$ $\;\mathrm{at}\;E_{Gate}=2.5\;J$	$MC_{Q}{\left(H\right)}_{E}$ $\;\mathrm{at}\;E_{Gate}=7\;J$
0.087	$9.4\times 10^{8}$ mS/cm^2^	101.5 ms/cm^2^
0.077	$4.3\times 10^{8}$ mS/cm^2^	43.4 mS/cm^2^
0.067	$1.8\times 10^{8}$ mS/cm^2^	18.3 mS/cm^2^

**Table 19:** Represents the values of quantum membrane conductance of extracellular protons that take the range between the two values calculated at $E_{Gate}=2.5$ J and $E_{Gate}=7$ J. The evaluation is made at n = 1, n = 2, and n = 4 and by setting $V_{m}=0.087$ V, L=1.5 m, and $D=10^{11}$ channels/cm^2^.

The Location of Gate n	$MC_{Q}{\left(H\right)}_{E}$ $\;\mathrm{at}\;E_{Gate}=2.5\;J$	$MC_{Q}{\left(H\right)}_{E}$ $\;\mathrm{at}\;E_{Gate}=7\;J$
1	$9.4\times 10^{8}$ mS/cm^2^	101.5 ms/cm^2^
2	$1.6\times 10^{7}$ mS/cm^2^	2.4 mS/cm^2^
4	$1.1\times 10^{6}$ mS/cm^2^	0.32 mS/cm^2^

**Table 20:** Represents the values of quantum membrane conductance of extracellular protons that take the range between the two values calculated at $E_{Gate}=2.5$ J and $E_{Gate}=7$ J. The evaluation is made at $D=10^{11}$ channels/cm^2^, $D=10^{10}$ channels/cm^2^, and $D=10^{9}$ channels/cm^2^ and by setting $V_{m}=0.087$ V, L=1.5 m, and n = 1.

The Density of Channels D (Channels/cm^2^)	$MC_{Q}{\left(H\right)}_{E}$ $\;\mathrm{at}\;E_{Gate}=2.5\;J$	$MC_{Q}{\left(H\right)}_{E}$ $\;\mathrm{at}\;E_{Gate}=7\;J$
$10^{11}$	$9.4\times 10^{8}$ mS/cm^2^	101.5 ms/cm^2^
$10^{10}$	$9.4\times 10^{7}$ mS/cm^2^	10.2 mS/cm^2^
$10^{9}$	$9.4\times 10^{6}$ mS/cm^2^	1.01 mS/cm^2^

**Table 21:** Represents the values of quantum membrane conductance of intracellular protons that take the range between the two values calculated at $E_{Gate}=1$ J and $E_{Gate}=7$ J. The evaluation is made at *L* = 1.5 m, *L* = 2 m, and *L* = 2.5 m and by setting $D=10^{11}$ channels/cm^2^.

The Gate Length *L* (m)	$MC_{Q}{\left(H\right)}_{I}$ $\;\mathrm{at}\;E_{Gate}=1\;J$	$MC_{Q}{\left(H\right)}_{I}$ $\;\mathrm{at}\;E_{Gate}=7\;J$
1.5	$3.6\times 10^{8}$ mS/cm^2^	0.041 ms/cm^2^
2	$1.6\times 10^{8}$ mS/cm^2^	$9.1\times 10^{-6}$ mS/cm^2^
2.5	$7.3\times 10^{7}$ mS/cm^2^	$2\times 10^{-9}$ mS/cm^2^

**Table 22:** Represents the values of quantum membrane conductance of intracellular protons that take the range between the two values calculated at $E_{Gate}=1$ J and $E_{Gate}=7$ J. The evaluation is made at $D=10^{11}$ channels/cm^2^, $D=10^{10}$ channels/cm^2^, and $D=10^{9}$ channels/cm^2^, and by setting L=1.5 m.

The Density of Channels D (Channels/cm^2^)	$MC_{Q}{\left(H\right)}_{I}$ $\;\mathrm{at}\;E_{Gate}=1\;J$	$MC_{Q}{\left(H\right)}_{I}$ $\;\mathrm{at}\;E_{Gate}=7\;J$
$10^{11}$	$3.6\times 10^{8}$ mS/cm^2^	0.041 ms/cm^2^
$10^{10}$	$3.6\times 10^{7}$ mS/cm^2^	$4.1\times 10^{-3}$ mS/cm^2^
$10^{9}$	$3.6\times 10^{6}$ mS/cm^2^	$4.1\times 10^{-4}$ mS/cm^2^

**Table 23:** Represents the values of quantum membrane conductance of extracellular sodium ions that take the range between the two values calculated at $E_{Gate}=2.5$ J and $E_{Gate}=7$ J. The evaluation is made at *L* = 1.5 m, *L* = 2 m, and *L* = 2.5 m and by setting $V_{m}=0.087$ V, $D=10^{11}$ channels/cm^2^, and n = 1.

The Gate Length *L* (m)	$MC_{Q}{\left(Na\right)}_{E}$ $\;\mathrm{at}\;E_{Gate}=2.5\;J$	$MC_{Q}{\left(Na\right)}_{E}$ $\;\mathrm{at}\;E_{Gate}=7\;J$
1.5	$4.5\times 10^{6}$ mS/cm^2^	$3.2\times 10^{-27}$ ms/cm^2^
2	$4.7\times 10^{5}$ mS/cm^2^	$2.9\times 10^{-39}$ mS/cm^2^
2.5	$4.9\times 10^{4}$ mS/cm^2^	$2.8\times 10^{-51}$ mS/cm^2^

**Table 24:** Represents the values of quantum membrane conductance of extracellular sodium ions that take the range between the two values calculated at $E_{Gate}=2.5$ J and $E_{Gate}=7$ J. The evaluation is made at $V_{m}=0.087$ V, $V_{m}=0.077$ V and $V_{m}=0.067$ V and by setting *L* = 1.5 m, $D=10^{11}$ channels/cm^2^, and n = 1.

The Membrane Potential *V_m_* (V)	$MC_{Q}{\left(Na\right)}_{E}$ $\;\mathrm{at}\;E_{Gate}=2.5\;J$	$MC_{Q}{\left(Na\right)}_{E}$ $\;\mathrm{at}\;E_{Gate}=7\;J$
0.087	$4.5\times 10^{6}$ mS/cm^2^	$3.2\times 10^{-27}$ ms/cm^2^
0.077	$1.1\times 10^{5}$ mS/cm^2^	$5.5\times 10^{-29}$ mS/cm^2^
0.067	$1.5\times 10^{3}$ mS/cm^2^	$9.1\times 10^{-31}$ mS/cm^2^

**Table 25:** Represents the values of quantum membrane conductance of extracellular sodium ions that take the range between the two values calculated at $E_{Gate}=2.5$ J and $E_{Gate}=7$ J. The evaluation is made at n = 1, n = 2, and n = 4 and by setting $V_{m}=0.087$ V, L=1.5 m, and $D=10^{11}$ channels/cm^2^.

The Location of Gate n	$MC_{Q}{\left(Na\right)}_{E}$ $\;\mathrm{at}\;E_{Gate}=2.5\;J$	$MC_{Q}{\left(Na\right)}_{E}$ $\;\mathrm{at}\;E_{Gate}=7\;J$
1	$4.5\times 10^{6}$ mS/cm^2^	$3.2\times 10^{-27}$ ms/cm^2^
2	$1.6\times 10^{-2}$ mS/cm^2^	$5.4\times 10^{-35}$ mS/cm^2^
4	$4.6\times 10^{-8}$ mS/cm^2^	$4\times 10^{-39}$ mS/cm^2^

**Table 26:** Represents the values of quantum membrane conductance of extracellular sodium ions that take the range between the two values calculated at $E_{Gate}=2.5$ J and $E_{Gate}=7$ J. The evaluation is made at $D=10^{11}$ channels/cm^2^, $D=10^{10}$ channels/cm^2^, and $D=10^{9}$ channels/cm^2^ and by setting $V_{m}=0.087$ V, L=1.5 m, and n = 1.

The Density of Channels D (Channels/cm^2^)	$MC_{Q}{\left(Na\right)}_{E}$ $\;\mathrm{at}\;E_{Gate}=2.5\;J$	$MC_{Q}{\left(Na\right)}_{E}$ $\;\mathrm{at}\;E_{Gate}=7\;J$
$10^{11}$	$4.5\times 10^{6}$ mS/cm^2^	$3.2\times 10^{-27}$ ms/cm^2^
$10^{10}$	$4.5\times 10^{5}$ mS/cm^2^	$3.2\times 10^{-28}$ mS/cm^2^
$10^{9}$	$4.5\times 10^{4}$ mS/cm^2^	$3.2\times 10^{-29}$ mS/cm^2^

**Table 27:** Represents the values of quantum membrane conductance of intracellular sodium ions that take the range between the two values calculated at $E_{Gate}=1$ J and $E_{Gate}=7$ J. The evaluation is made at *L* = 1.5 m, *L* = 2 m, and *L* = 2.5 m and by setting $D=10^{11}$ channels/cm^2^.

The Gate Length *L* (m)	$MC_{Q}{\left(Na\right)}_{I}$ $\;\mathrm{at}\;E_{Gate}=1\;J$	$MC_{Q}{\left(Na\right)}_{I}$ $\;\mathrm{at}\;E_{Gate}=7\;J$
1.5	$4.6\times 10^{4}$ mS/cm^2^	$2.2\times 10^{-43}$ ms/cm^2^
2	$1.1\times 10^{3}$ mS/cm^2^	$8.5\times 10^{-61}$ mS/cm^2^
2.5	24 mS/cm^2^	$3.3\times 10^{-78}$ mS/cm^2^

**Table 28:** Represents the values of quantum membrane conductance of intracellular sodium ions that take the range between the two values calculated at $E_{Gate}=1$ J and $E_{Gate}=7$ J. The evaluation is made at $D=10^{11}$ channels/cm^2^, $D=10^{10}$ channels/cm^2^, and $D=10^{9}$ channels/cm^2^, and by setting L=1.5 m.

The Density of Channels D (Channels/cm^2^)	$MC_{Q}{\left(Na\right)}_{I}$ $\;\mathrm{at}\;E_{Gate}=1\;J$	$MC_{Q}{\left(Na\right)}_{I}$ $\;\mathrm{at}\;E_{Gate}=7\;J$
$10^{11}$	$4.6\times 10^{4}$ mS/cm^2^	$2.2\times 10^{-43}$ ms/cm^2^
$10^{10}$	$4.6\times 10^{3}$ mS/cm^2^	$2.2\times 10^{-44}$ mS/cm^2^
$10^{9}$	$4.6\times 10^{2}$ mS/cm^2^	$2.2\times 10^{-45}$ mS/cm^2^

**Table 29:** Represents the values of point of curving for protons $E_{cur}(H)$, the membrane potential at $E_{Gate}=1$ J, and the average rate of depolarization. The evaluation is made at three different values of $pH_{E}$ ($pH_{E}=7.4$, $pH_{E}=7$, and $pH_{E}=6.5$) and by setting L=1.5 m, $D=10^{11}$ channels/cm^2^, and n = 1.

$pH_{E}$	$\mathrm{Point}\;\mathrm{of}\;\mathrm{Curving}\;E_{cur}(H)\;(J)$	$\mathrm{Membrane}\;\mathrm{Potential}\;\mathrm{at}\;E_{Gate}=1\;J$	Average Rate of Depolarization R(H) (V/J)
7.4	5.92	0.014 V	$1.5\times 10^{-2}$
7	6.22	0.014 V	$1.4\times 10^{-2}$
6.5	6.61	0.014 V	$1.3\times 10^{-2}$

**Table 30:** Represents the values of point of curving for protons $E_{cur}(H)$, the membrane potential at $E_{Gate}=1$ J, and the average rate of depolarization. The evaluation is made at three different values of gate length (L=1.5 m, L=2 m, and L=2.5 m) and by setting $pH_{E}=7.4$, $D=10^{11}$ channels/cm^2^, and n = 1.

The Gate Length *L* (m)	$\mathrm{Point}\;\mathrm{of}\;\mathrm{Curving}\;E_{cur}(H)\;(J)$	$\mathrm{Membrane}\;\mathrm{Potential}\;\mathrm{at}\;E_{Gate}=1\;J$	Average Rate of Depolarization R(H) (V/J)
1.5	5.92	0.014 V	$1.5\times 10^{-2}$
2	4.83	0.01 V	$2\times 10^{-2}$
2.5	4.24	0.0084 V	$2.4\times 10^{-2}$

**Table 31:** Represents the values of point of curving for protons $E_{cur}(H)$, the membrane potential at $E_{Gate}=1$ J, and the average rate of depolarization. The evaluation is made at three different values of channels density ($D=10^{11}$ channels/cm^2^, $D=10^{10}$ channels/cm^2^, and $D=10^{9}$ channels/cm^2^) and by setting $pH_{E}=7.4$, L=1.5 m, and n = 1.

The Density of Channels D (Channels/cm^2^)	$\mathrm{Point}\;\mathrm{of}\;\mathrm{Curving}\;E_{cur}(H)\;(J)$	$\mathrm{Membrane}\;\mathrm{Potential}\;\mathrm{at}\;E_{Gate}=1\;J$	Average Rate of Depolarization R(H) (V/J)
$10^{11}$	5.92	0.014 V	$1.5\times 10^{-2}$
$10^{10}$	5.2	0.014 V	$1.7\times 10^{-2}$
$10^{9}$	4.54	0.014 V	$2\times 10^{-2}$

**Table 32:** Represents the values of point of curving for protons $E_{cur}(H)$, the membrane potential at $E_{Gate}=1$ J, and the average rate of depolarization. The evaluation is made at three different values of gate location (n = 1, n = 2, and n = 4) and by setting $pH_{E}=7.4$, L=1.5 m, and $D=10^{11}$ channels/cm^2^.

The Location of Gate n	$\mathrm{Point}\;\mathrm{of}\;\mathrm{Curving}\;E_{cur}(H)\;(J)$	$\mathrm{Membrane}\;\mathrm{Potential}\;\mathrm{at}\;E_{Gate}=1\;J$	Average Rate of Depolarization R(H) (V/J)
1	5.92	0.014 V	$1.5\times 10^{-2}$
2	4.65	0.022 V	$1.8\times 10^{-2}$
4	3.97	0.031 V	$1.9\times 10^{-2}$

**Table 33:** Represents the values of point of curving for sodium ions $E_{cur}(Na)$, the zero membrane potential and the corresponding $E_{Gate}$, and the average rate of depolarization. The evaluation is made at three different values of gate length (L=1.5 m, L=2 m, and L=2.5 m) and by setting $D=10^{11}$ channels/cm^2^, and n = 1.

The Gate Length *L* (m)	$\mathrm{Point}\;\mathrm{of}\;\mathrm{Curving}\;E_{cur}(Na)\;(J)$	$\mathrm{Zero}\;\mathrm{Membrane}\;\mathrm{Potential}\;\mathrm{and}\;\mathrm{the}\;\mathrm{Corresponding}\;E_{Gate}$	Average Rate of Depolarization R(Na) (V/J)
1.5	3.62	0 V at $E_{Gate}=1.33$ J	$3.8\times 10^{-2}$
2	3.25	0 V at $E_{Gate}=1.16$ J	$4.1\times 10^{-2}$
2.5	3.03	0 V at $E_{Gate}=1.06$ J	$4.4\times 10^{-2}$

**Table 34:** Represents the values of point of curving for sodium ions $E_{cur}(Na)$, the zero membrane potential and the corresponding $E_{Gate}$, and the average rate of depolarization. The evaluation is made at three different values of channels density ($D=10^{11}$ channels/cm^2^, $D=10^{10}$ channels/cm^2^, and $D=10^{9}$ channels/cm^2^) and by setting L=1.5 m, and n = 1.

The Density of Channels D (Channels/cm^2^)	$\mathrm{Point}\;\mathrm{of}\;\mathrm{Curving}\;E_{cur}(Na)\;(J)$	$\mathrm{Zero}\;\mathrm{Membrane}\;\mathrm{Potential}\;\mathrm{and}\;\mathrm{the}\;\mathrm{Corresponding}\;E_{Gate}$	Average Rate of Depolarization R(Na) (V/J)
$10^{11}$	3.62	0 V at $E_{Gate}=1.33$ J	$3.76\times 10^{-2}$
$10^{10}$	3.5	0 V at $E_{Gate}=1.26$ J	$3.84\times 10^{-2}$
$10^{9}$	3.38	0 V at $E_{Gate}=1.19$ J	$3.9\times 10^{-2}$

**Table 35:** Represents the values of point of curving for sodium ions $E_{cur}(Na)$, the zero membrane potential and the corresponding $E_{Gate}$, and the average rate of depolarization. The evaluation is made at three different values of gate location (n= 1, n = 2, and n = 4) and by setting L=1.5 m, and $D=10^{11}$ channels/cm^2^.

The Location of Gate n	$\mathrm{Point}\;\mathrm{of}\;\mathrm{Curving}\;E_{cur}(Na)\;(J)$	$\mathrm{Zero}\;\mathrm{Membrane}\;\mathrm{Potential}\;\mathrm{and}\;\mathrm{the}\;\mathrm{Corresponding}\;E_{Gate}$	Average Rate of Depolarization R(Na) (V/J)
1	3.62	0 V at $E_{Gate}=1.33$ J	$3.8\times 10^{-2}$
2	2.62	0 V at $E_{Gate}=1.33$ J	$6.7\times 10^{-2}$
4	2.1	0 V at $E_{Gate}=1.33$ J	0.11

**Table 36:** Represents the values of membrane potential that take the range between the two values calculated at $pH_{E}=7.4$ and at $pH_{E}=5$ under the influence of classical transport through open sodium channels. The evaluation is made at three different setting values of channels density ($D=10^{11}$ channels/cm^2^, $D=10^{10}$ channels/cm^2^, $D=10^{9}$ channels/cm^2^) and by setting the permeability ratio $P_{H}/P_{Na}=252$, and the conductance of single channel $C_{Na}=17.3\times 10^{-12}$ S.

The Density of Channels (Channels/cm^2^)	$\mathrm{Membrane}\;\mathrm{Potential}\;(V)\;\mathrm{at}\;pH_{E}=7.4$	$\mathrm{Membrane}\;\mathrm{Potential}\;(V)\;\mathrm{at}\;pH_{E}=5$
$10^{11}$	0.067	0.062
$10^{10}$	0.08	0.062
$10^{9}$	0.086	0.064

## Data Availability

Not applicable.
